# Astrocyte-intrinsic signaling of chitinase-like protein CHI3L1 drives inflammation and amplifies demyelination in neuromyelitis optica

**DOI:** 10.1172/JCI195506

**Published:** 2026-01-02

**Authors:** Huiming Xu, Wei Jiang, Li Xu, Haoyang Li, Xin Yang, Fan Zhu, Pengyan He, Yanna Song, Yuhan Li, Yu-Wen Alvin Huang, Wei Qiu, Changyong Tang

**Affiliations:** 1 Department of Neurology, Third Affiliated Hospital of Sun Yat-Sen University, Guangzhou, Guangdong, China.; 2 Department of Molecular Biology, Cell Biology, and Biochemistry, Center for Translational Neuroscience, Carney Institute for Brain Science, Brown University, Providence, Rhode Island, USA.

**Keywords:** Autoimmunity, Neuroscience, Biomarkers, Mouse models, Therapeutics

## Abstract

Neuromyelitis optica (NMO) is an autoimmune disorder characterized by autoantibodies against the astrocyte water channel aquaporin-4 (AQP4) that cause demyelination in the optic nerves and spinal cord. How astrocytopathy leads to myelination deficits remains unclear. Chitinase-3–like protein 1 (CHI3L1, also known as YKL-40) is predominantly secreted by activated astrocytes, serves as a robust NMO biomarker, and plays a role in immune responses, but how it is induced and shapes astrocyte activation in NMO is not well defined. Using ex vivo and in vivo NMO mouse models together with mice with astrocyte-specific CHI3L1 knockout, we demonstrated that CHI3L1 directly contributed to demyelinating lesions elicited by AQP4 autoantibody–activated astrocytes. With complementary in vitro assays and inducible transgenic lines, we uncovered an astrocyte-intrinsic cascade in which AQP4 autoantibody exposure activated STAT3, which in turn drove CHI3L1 expression and secretion. Secreted CHI3L1 then engaged the astrocytic receptor RAGE in an autocrine manner, activating downstream NF-κB signaling that drove proinflammatory gliosis and damaged myelination. Pharmacological blockade of this pathway in NMO models rescued demyelinating pathology and improved motor function. These findings reveal an astrocyte-intrinsic CHI3L1 pathway that contributed to demyelination in NMO and identify actionable therapeutic targets.

## Abstract 2

Astrocyte-secreted CHI3L1, an inflammatory biomarker and pathogenic mediator, drives neuromyelitis optica demyelination via a specified receptor signaling mechanism. Pharmacological inhibitors reduce damage, offering treatment hope.

## Introduction

Neuromyelitis optica (NMO) spectrum disorder is an autoimmune inflammatory demyelinating disease characterized by severe attacks of central nervous system (CNS) inflammation, predominantly affecting the spinal cord and optic nerves. The disease is mediated by autoantibodies against aquaporin-4 (AQP4), a water channel protein expressed on astrocytes. Detection of AQP4-specific autoantibodies (AQP4-IgG, also known as NMO-IgG) in patients’ serum is crucial for diagnosing NMO and distinguishes it from multiple sclerosis (MS) (1). Although the pathogenic role of AQP4-IgG in NMO is supported by histological findings and in vitro and in vivo studies, the mechanisms by which astrocyte damage leads to oligodendrocyte dysfunction and demyelination remain incompletely understood. Particularly, how astrocyte activation by autoantibodies contributes to myelination deficits — the major source of clinical manifestations — requires further elucidation.

Chitinase-3–like protein 1 (CHI3L1), also known as YKL-40 in humans and breast regression protein 39 (BRP-39) in mice, is a member of the chitinase protein family that retains a conserved chitin-binding domain but lacks enzymatic activity (2–4). CHI3L1 is predominantly secreted by activated astrocytes in the CNS (5–7) and has been strongly associated with neuroimmune disorders such as NMO (8–10) and MS (11), as well as neurodegenerative conditions like Alzheimer’s disease (12, 13). Beyond its peripheral roles in pathogen defense and injury response, CHI3L1 is implicated in neuroinflammatory processes and astrocyte activation within the CNS (5–7, 14). Our recent research has demonstrated that CHI3L1 functions beyond a biomarker in NMO pathogenesis, revealing its role in suppressing hippocampal neurogenesis — a process that can be targeted to restore cognitive decline in NMO mouse models (15). However, the mechanisms by which CHI3L1 is induced in astrocytes targeted by AQP4 autoantibodies, and how it subsequently modulates astrocyte activation and inflammatory damage, are not well understood.

In this study, we employed ex vivo and in vivo mouse models of NMO and showed that CHI3L1 directly contributes to demyelinating lesions driven by AQP4 autoantibody–activated astrocytes. Using inducible, astrocyte-specific knockouts of CHI3L1 and related effectors, we define an astrocyte-intrinsic cascade mediating demyelination and neurological symptoms in NMO. Specifically, we found that AQP4 autoantibody attack activates signal transducer and activator of transcription 3 (STAT3) in astrocytes, leading to CHI3L1 expression and secretion. The secreted CHI3L1 then engages the receptor for advanced glycation end products (RAGE) on astrocytes in an autocrine manner, activating the downstream nuclear factor-κB (NF-κB) signaling pathway. This sequence drives proinflammatory gliosis and damages myelination. Importantly, pharmacological blockade at these checkpoints rescues demyelination and motor deficits. Thus, a CHI3L1-centered astrocytic pathway emerges as a therapeutic target for NMO and related demyelinating disorders.

## Results

### CHI3L1 contributes to demyelination and neurological deficits in multiple NMO models.

To create NMO models for studying the effect of astrocyte activation by AQP4 autoantibodies on myelination, we first purified total IgG from the serum of NMO patients who were seropositive for AQP4-IgG (AQP4-IgG) and from healthy controls (Ctrl-IgG), following our established protocol (16) (Supplemental Figure 1A and Table 1; supplemental material available online with this article; https://doi.org/10.1172/JCI195506DS1). This method, independently documented, reproduces key pathological features of NMO (15, 17, 18).

**Figure 1:**
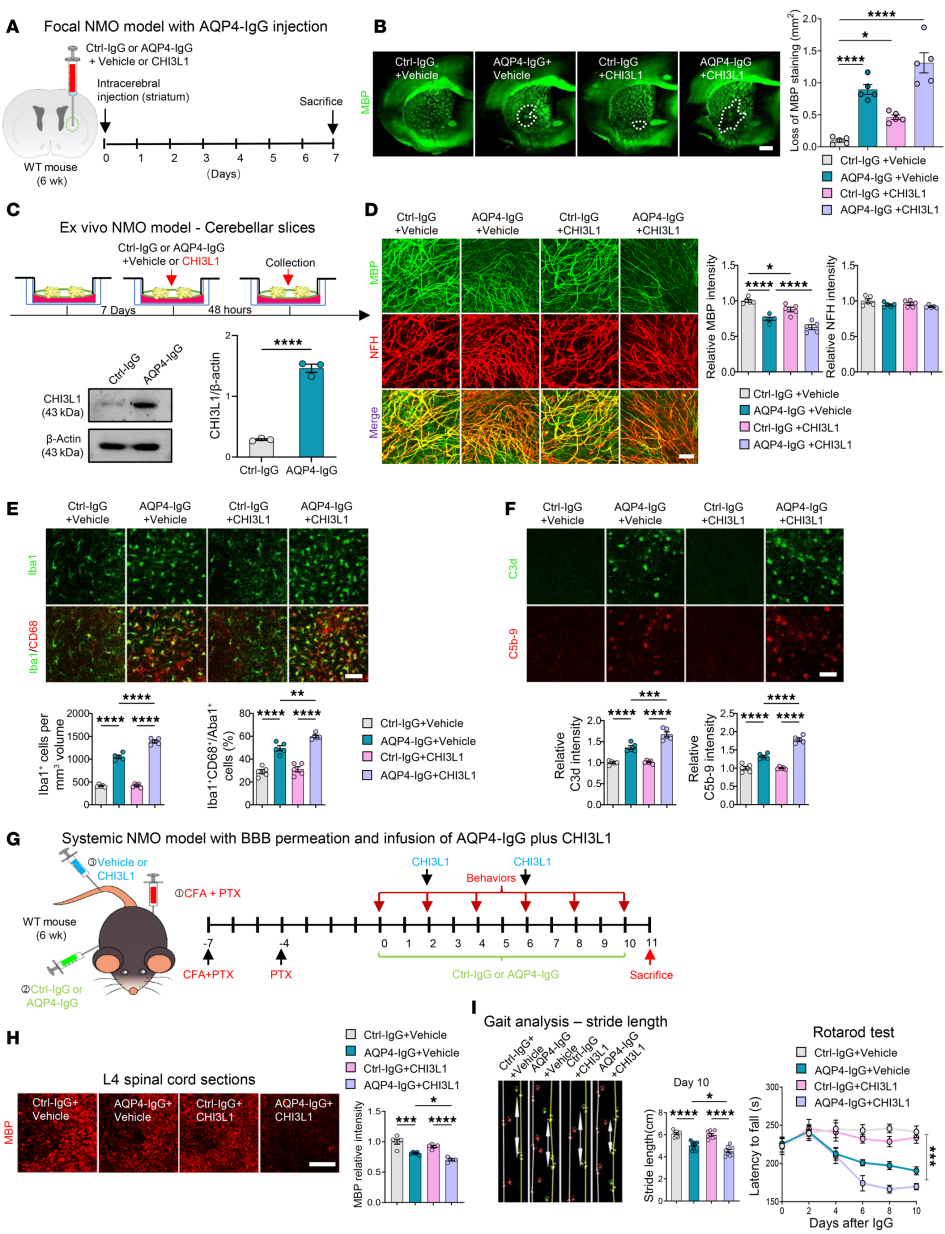
CHI3L1 amplifies demyelination and inflammation in NMO models driven by human AQP4 autoantibodies. (**A**) Focal NMO model: stereotaxic infusion into mouse striatum of AQP4-IgG or control IgG (Ctrl-IgG) with or without CHI3L1 (200 ng). (**B**) Confocal images showing striatal demyelinating lesions; myelin basic protein (MBP) loss areas (white dashed lines) quantified across groups. Scale bar: 500 μm; *n* = 5 mice per group (3 sections per mouse). (**C**) Ex vivo model: organotypic cerebellar slices (P7, 7 days in vitro) treated 48 hours with AQP4-IgG or Ctrl-IgG with or without CHI3L1 (100 ng/mL); immunostaining for NMO markers and immunoblot validation of CHI3L1 induction (*n* = 3 biological replicates per group). (**D**) Ex vivo demyelination quantified by MBP signal (normalized to Ctrl-IgG + vehicle = 1.0); neurofilament-heavy (NFH) used to assess axonal process integrity. Scale bar: 300 μm; *n* = 5 slices per group (3 sections per slice). (**E**) Ex vivo microglial activation quantified as Iba1^+^ total and Iba1^+^CD68^+^ activated microglia. Scale bar: 100 μm; *n* = 5 slices per group. (**F**) Ex vivo complement activation: C3d levels and membrane attack complex (C5b-9), normalized to Ctrl-IgG + vehicle = 1.0. Scale bar: 100 μm; *n* = 5 mice per group (3 sections per mouse). (**G**) Systemic NMO model in vivo: blood-brain/blood-spinal barrier disruption with CFA (s.c.) and pertussis toxin (PTX) (i.p.), followed by daily i.p. AQP4-IgG or Ctrl-IgG; CHI3L1 (1 μg/mouse) or vehicle given i.v. at indicated times. (**H**) Lumbar spinal cord (L4) demyelination quantified by MBP intensity normalized to Ctrl-IgG + vehicle. Scale bar: 100 μm; *n* = 5 mice per group (3 sections per mouse). (**I**) Motor deficits: gait (stride length) and rotarod latency in systemic model (*n* = 8 mice per group). Statistics: Mean ± SEM. Student’s *t* test (**C**); 2-way ANOVA for rotarod latency (**I**); all other comparisons by 1-way ANOVA with Tukey’s post hoc test or Welch’s ANOVA with Dunnett’s T3 test for unequal variances. Non-significant comparisons are not shown. *P < 0.05; **P < 0.01; ***P < 0.001; ****P < 0.0001.

Based on our passive-transfer mouse model of NMO — established by intracerebral stereotaxic infusion of human autoantibodies to create a focal NMO model (15) — we injected Ctrl-IgG, AQP4-IgG, or AQP4-IgG plus recombinant CHI3L1 into the striatum (Figure 1A), a brain region that frequently exhibits demyelinating pathology associated with motor dysfunction in NMO patients (19, 20). To anchor subsequent analyses, we performed a longitudinal assessment (1–28 days after injection) of the focal striatal lesions (Supplemental Figure 1B). Oligodendroglial loss and demyelination acutely emerged by day 1 and peaked at day 3, while microgliosis peaked at day 7, followed by partial recovery by day 14 and near baseline by day 28; accordingly, we focused on the 7-day time point for downstream experiments.

Consistent with NMO histopathological findings, we observed a marked loss of AQP4 expression due to internalization and degradation (1, 17, 18, 21), along with a significant increase in CHI3L1 expression at the AQP4-IgG injection site within the striatum compared with Ctrl-IgG injection within 7 days after injection (Supplemental Figure 1, C and D). Importantly, we noted a concomitant loss of myelin signals at the AQP4-IgG injection site (Figure 1B), demonstrating demyelination caused by AQP4-IgG as seen in NMO. Strikingly, CHI3L1 alone without AQP4-IgG caused moderate yet significant demyelination pathology, and coadministration of CHI3L1 with AQP4-IgG exacerbated the demyelination pathology (Figure 1B), suggesting that CHI3L1 actively contributes to demyelination in NMO.

To further investigate the effects of CHI3L1 on myelin integrity, we developed an ex vivo organotypic culture model of NMO using vibratome-cut cerebellar slices — a method widely used to study myelination and white matter diseases (16) — and treated them with Ctrl-IgG, AQP4-IgG, or AQP4-IgG plus CHI3L1 (Figure 1C). Within 48 hours of treatment with AQP4-IgG, we confirmed induction of CHI3L1 expression and observed a loss of myelin signals. The demyelination was further aggravated by coadministration of CHI3L1 (Figure 1D). Given that CHI3L1 is a key modulator in innate immunity (3), we examined microglial status and inflammatory activity. We observed that AQP4-IgG treatment not only increased the number of microglia but also enhanced microglial activation (Figure 1E) and the resultant innate immune response, marked by increased complement activation and formation of the membrane attack complex (Figure 1F). Notably, under this acute treatment setting, neuronal axonal bundles were spared from AQP4-IgG– and/or CHI3L1-induced damage (Figure 1D), supporting the notion that myelin is vulnerable in NMO at the early stage, leading to loss of neuronal protection. These findings establish the utility of the focal and ex vivo NMO models we developed and provide insight into the active role of CHI3L1 in NMO demyelination pathogenesis.

To better mimic the functional consequences of demyelination caused by AQP4-IgG, we used a systemic NMO model involving the passive transfer of AQP4-IgG to mice via circulation (Figure 1G). This was facilitated by chemical disruption of the blood-brain barrier (BBB) and blood–spinal cord barrier to allow AQP4-IgG penetration into the CNS (16, 22). Comparable to the more complex active-immunization model (23, 24), where animals are immunized with recombinant AQP4, this systemic model has been demonstrated to display characteristic spinal cord demyelination lesions and motor dysfunction, such as disturbed gait and motor coordination (16, 22).

To elucidate the contribution of CHI3L1 to spinal cord demyelination and motor deficits, we systemically administered recombinant CHI3L1 to mice in the systemic NMO model. We performed motor function behavioral assays followed by histopathological examinations of lumbar spinal cord sections (Figure 1G). We first confirmed that passive transfer of AQP4-IgG, compared with Ctrl-IgG, induced the characteristic pathological feature of loss of AQP4 expression along the astrocyte end-feet lining the vasculature (Supplemental Figure 2A). We demonstrated loss of myelination in these spinal cord lesions (Figure 1H), accompanied by activation of astrocytes and microglia (Supplemental Figure 2A). The inflammatory features were further corroborated by analyses of lumbar spinal cord tissues showing induction of an array of proinflammatory cytokines (Supplemental Figure 2, B and C). Importantly, all these pathological features were aggravated by the coadministration of CHI3L1 (Figure 1H and Supplemental Figure 2, A–C).

Moreover, over the experimental course of 2 weeks, we observed loss of lumbar spinal cord neurons, particularly within the ventral horn region, in the systemic NMO model mice (Supplemental Figure 2A). Consistent with neuronal dysfunction, behavioral tests, including gait analysis for foot stride length and the rotarod test for motor coordination, revealed significant deficits in mice of the systemic NMO model (Figure 1I). Coadministration of CHI3L1 significantly worsened neuronal loss within the lumbar spinal cord as well as the behavioral deficits (Supplemental Figure 2A).

Across models, we also tested CHI3L1 alone (Ctrl-IgG + CHI3L1). In the focal striatal and ex vivo cerebellar slice paradigms, CHI3L1 by itself produced a moderate but significant reduction in myelin basic protein (MBP) relative to vehicle (Ctrl-IgG + vehicle), whereas AQP4-IgG + CHI3L1 yielded the greatest demyelination (Figure 1, B and D). In the systemic model, CHI3L1 alone showed only a subtle, non-significant trend toward reduced myelin yet robustly increased inflammatory readouts (Supplemental Figure 2, A–C) and exacerbated AQP4-IgG–induced demyelination and inflammation (Figure 1H). Taken together, these results support context-dependent pathogenicity: CHI3L1 can impair myelin integrity in localized/controlled settings, while in vivo it functions chiefly as an amplifier of AQP4-IgG–triggered astrocyte injury within a complex immune milieu (e.g., BBB dynamics, ligand clearance, compensatory glial responses).

These in vivo observations prompted us to ask whether CHI3L1 also directly perturbs the oligodendrocyte lineage; we therefore turned to primary OPC assays to test cell-autonomous effects on proliferation, differentiation, and myelin ensheathment. Primary mouse oligodendrocyte precursor cells (OPCs) were isolated from juvenile wild-type brains (25), cultured, and exposed to CHI3L1 to mimic astrocyte-derived ligand. CHI3L1 significantly reduced OPC proliferation, limiting the self-renewing pool that supplies myelinating oligodendrocytes (Supplemental Figure 2D). We then induced OPC differentiation in vitro (25) and applied CHI3L1 at the pre-myelinating stage: CHI3L1 lowered myelin protein expression in mature oligodendrocytes without increasing cell death (Supplemental Figure 2, E and F). Finally, using a nanofiber coculture system that models axonal ensheathment (26), CHI3L1-treated oligodendrocytes showed impaired wrapping of synthetic fibers, indicating defective initiation of myelination (Supplemental Figure 2G). Together, these experiments demonstrate a cell-autonomous, lineage-directed inhibitory effect of CHI3L1 on oligodendroglial development — dampening OPC proliferation and hindering myelinating differentiation.

Collectively, our findings across the focal, ex vivo, and systemic NMO models strongly support the active role of CHI3L1 in promoting demyelination and consequent motor dysfunction in NMO. This underscores the critical importance of CHI3L1 in NMO pathogenesis and highlights its potential as a therapeutic target for mitigating demyelination and neurological deficits associated with the disease.

### Ablation of astrocyte-secreted CHI3L1 alleviates demyelination and motor dysfunction in NMO models.

To directly test our hypothesis that targeting CHI3L1 secreted from AQP4-IgG–induced astrocyte activation can alleviate demyelination lesions and motor dysfunction in NMO, we used a monoclonal anti-CHI3L1 neutralizing antibody that we previously demonstrated to block CHI3L1 actions in the brain (15). We first employed the ex vivo model using cerebellar slice cultures, administering anti-CHI3L1 or a control IgG in combination with treatments of Ctrl-IgG or AQP4-IgG (Supplemental Figure 3A). Coadministration of anti-CHI3L1 significantly rescued the myelin loss induced by AQP4-IgG (Supplemental Figure 3B), reduced microglial activation, and diminished innate immune responses (Supplemental Figure 3C). These findings suggest that neutralizing secreted CHI3L1 can alleviate proinflammatory neurotoxicity in NMO.

**Figure 2:**
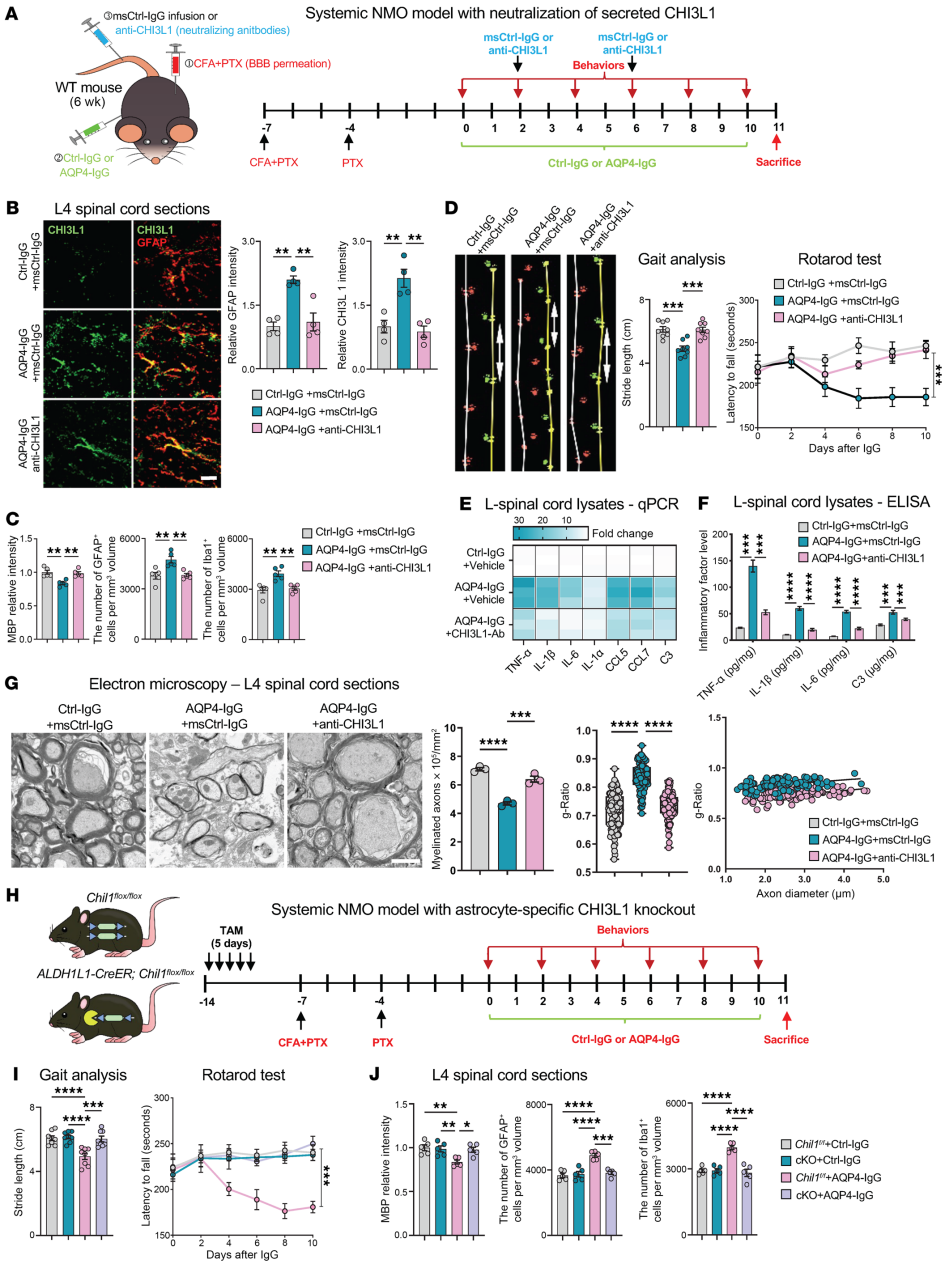
Astrocyte-secreted CHI3L1 is required for NMO-related demyelination, glial activation, and motor deficits. (**A**) Systemic NMO model: BBB/blood–spinal cord barrier disruption with CFA (s.c.) and PTX (i.p.), followed by daily i.p. AQP4-IgG or Ctrl-IgG; anti-CHI3L1 (10 μg, i.v.) or control IgG (msCtrl-IgG) administered at indicated times. ms, mouse. (**B**) L4 spinal cord confocal images showing that anti-CHI3L1 reduces AQP4-IgG–induced CHI3L1 upregulation and astrocyte activation (GFAP). Quantified GFAP and CHI3L1 signals normalized to Ctrl-IgG + vehicle = 1.0. Scale bar: 20 μm; *n* = 4 mice per group (3 sections per mouse). (**C**) Anti-CHI3L1 mitigates demyelination and gliosis: MBP intensity and densities of GFAP^+^ astrocytes and Iba1^+^ microglia in L4 sections. *n* = 5 mice per group (3 sections per mouse). (**D**) Motor function: anti-CHI3L1 improves stride length and rotarod latency in systemic NMO mice. *n* = 8 per group. (**E**) Quantitative PCR (qPCR) heatmap of NF-κB–regulated cytokines (TNF-α, IL-1β, IL-6, IL-1α, CCL5, CCL7, C3) in lumbar cord after CHI3L1 neutralization, normalized to Ctrl-IgG + vehicle. *n* = 3 per group. (**F**) ELISA of secreted cytokines in lumbar cord lysates with anti-CHI3L1 versus msCtrl-IgG. *n* = 3 per group. (**G**) Electron microscopy of L4 white matter showing preservation of myelin with anti-CHI3L1. Scale bar: 2 μm; *n* = 3 animals per group. Quantified myelinated-axon density (normalized to Ctrl-IgG + msCtrl-IgG = 1.0) and g-ratio; 150 axons per group from 3 animals. (**H**) Conditional astrocyte-specific CHI3L1 knockout (Chil1 cKO): *Chil1^fl/fl^* × ALDH1L1-CreERT2, tamoxifen-induced; subjected to systemic NMO paradigm, followed by behavioral testing and histopathology. (**I**) Chil1 cKO improves gait and rotarod performance versus floxed controls under AQP4-IgG. *n* = 8 per group. (**J**) Chil1 cKO reduces demyelination and gliosis: MBP intensity and GFAP^+^Iba1^+^ cell densities in L4 sections. *n* = 5 per group (3 sections per mouse). Statistics: Mean ± SEM. Rotarod latency analyzed by 2-way ANOVA (**D** and **I**). All other bar graphs: 1-way ANOVA with Tukey’s post hoc test or Welch’s ANOVA with Dunnett’s T3 test for unequal variances. Non-significant comparisons are not shown. *P < 0.05; **P < 0.01; ***P < 0.001; ****P < 0.0001.

To extend these findings in vivo, we used the systemic NMO model and administered anti-CHI3L1 or control IgG, followed by behavioral testing and lumbar cord histology (Figure 2A). Anti-CHI3L1 markedly reduced astrocyte activation and CHI3L1 expression (Figure 2B), suggesting that blocking CHI3L1 function exerts an antiinflammatory effect on astrocytes. This reduction in astrocyte activation likely attenuates the downstream inflammatory cascade contributing, to demyelination. Moreover, in systemic NMO mice, anti-CHI3L1 treatment decreased myelin loss, preserved myelinated axons (Figure 2C; assayed by MBP staining), and reduced microgliosis, accompanied by prevention of ventral horn neuronal loss in the lumbar spinal cord (Supplemental Figure 3D). These tissue-level benefits aligned with improved gait and motor coordination (Figure 2D), and attenuated the induction of proinflammatory cytokines and chemokines (Figure 2, E and F). Electron microscopy revealed disruptions of the myelin sheath with relatively preserved axonal processes in NMO mice, which were improved by anti-CHI3L1 treatment (Figure 2G; assayed by electron microscopy).

Additionally, we used a focal NMO rat model that enabled MRI imaging for the analysis of white matter changes akin to clinical examinations in NMO patients. Young adult wild-type rats received stereotaxic injections of Ctrl-IgG or AQP4-IgG into the striatum as previously described (Figure 1A), along with coadministration of anti-CHI3L1 or control IgG. The rats underwent MRI scans before sacrifice for brain tissue collection (Supplemental Figure 3E). AQP4-IgG injection induced CHI3L1 expression (Supplemental Figure 3F) and reproduced characteristic white matter lesions observed in NMO, evidenced by hyperintense signals on MRI indicative of demyelination (Supplemental Figure 3G). Importantly, coadministration of anti-CHI3L1 significantly lessened the white matter lesions in the focal NMO rat model (Supplemental Figure 3G), demonstrating the efficacy of anti-CHI3L1 in attenuating demyelination detectable by clinical imaging modalities. Together, our findings across 3 independent NMO models support the neuroprotective effect of blocking secreted CHI3L1 functions.

We next sought to further ascertain the role of astrocyte-secreted CHI3L1 in NMO pathogenesis, hypothesizing that deletion of CHI3L1 in astrocytes would reverse demyelination and motor deficits. We generated a strain of mice with conditional astrocyte-specific CHI3L1 knockout (Chil1 cKO) by breeding our CHI3L1-floxed mice (*Chil1^fl/fl^*) (15) with ALDH1L1-CreERT2 mice to allow tamoxifen-induced deletion (Supplemental Figure 4A). Using the systemic NMO model, we treated the conditional-knockout mice and control CHI3L1-floxed mice with tamoxifen, followed by administration of AQP4-IgG or Ctrl-IgG. AQP4-IgG or Ctrl-IgG were administrated i.p. peripherally and, as the BBB was temporarily disrupted by CFA+PTX, were able to penetrate into brain (Figure 2H). Systemic administration of AQP4-IgG induced characteristic pathological changes in control mice, including gait disturbance (Supplemental Figure 4B and Figure 2I), impaired motor coordination (Figure 2I), loss of ventral horn neurons (Supplemental Figure 4C), demyelination accompanied by activation of astrocytes and microglia (Figure 2J), and induction of proinflammatory activities within the lumbar spinal cord (Supplemental Figure 4, D and E). Strikingly, ablation of astrocytic CHI3L1 significantly rescued all these pathological changes caused by systemic delivery of AQP4-IgG (Figure 2, H–J, and Supplemental Figure 4, B–E).

Targeting astrocyte-secreted CHI3L1 — which activates microglia and impairs myelination — may mitigate NMO pathology and neurological deficits triggered by anti-AQP4 IgG. Because blocking secreted CHI3L1 reduced both CHI3L1 induction and astrogliosis across models, CHI3L1 likely acts autocrinely on astrocytes. Defining this astrocyte-intrinsic mechanism will sharpen our understanding of NMO and guide targeted therapies for primary astrocytopathies.

### Autocrine CHI3L1 activation of NF-κB signaling in astrocytes mediates inflammatory activation by AQP4 antibody attack.

CHI3L1 has been demonstrated to be a key player in intercellular interactions during inflammatory and immune responses, acting in a paracrine manner to facilitate glia-to-glia communication (e.g., microglial activation) (5) and glia-to-neuron signaling (e.g., inhibition of neuronal function) (13, 15). Our findings suggest that astrocyte-secreted CHI3L1 can also act in an autocrine manner on astrocytes themselves, influencing their cellular states — a notion of particular importance for primary astrocytopathies such as NMO. Recognizing the potential therapeutic implications of this autocrine signaling, we aimed to decipher the molecular mechanisms of the intrinsic function of CHI3L1 in astrocyte activation. To this end, we employed a reductionist approach using primary cultures of mouse astrocytes to delineate the responsible signaling cascades.

**Figure 3:**
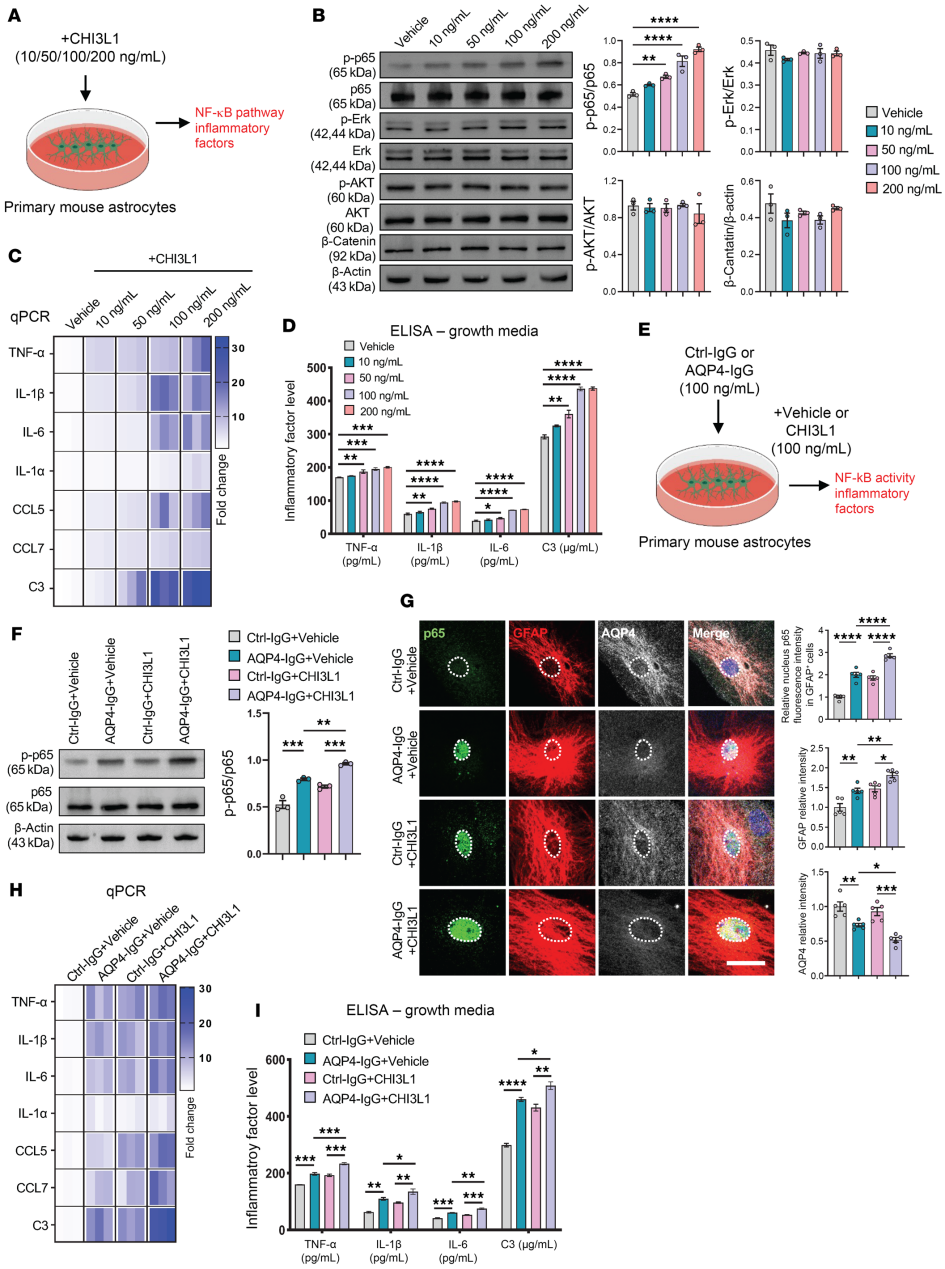
CHI3L1 activates astrocytic NF-κB and potentiates AQP4-IgG–evoked proinflammatory responses. (**A**) In vitro paradigm: primary mouse astrocytes exposed to recombinant human CHI3L1 at graded doses to assess cell-intrinsic signaling. (**B**) CHI3L1 (10–200 ng/mL, 4 hours) selectively increases NF-κB p65 phosphorylation without activating ERK, AKT, or β-catenin; immunoblot densitometry shown as phospho/total ratios (*n* = 3 experiments). (**C**) CHI3L1 dose-dependently induces NF-κB target transcripts (TNF-α, IL-1β, IL-6, IL-1α, CCL5, CCL7, C3) at 24 hours; heatmap normalized to vehicle (0 ng/mL) (*n* = 3). (**D**) Corresponding cytokine secretion (TNF-α, IL-1β, IL-6, C3) measured by ELISA from conditioned media after 24 hours of CHI3L1 (10–200 ng/mL) (*n* = 3). (**E**) Costimulation design: astrocytes treated with Ctrl-IgG or AQP4-IgG (100 ng/mL) with or without CHI3L1 (100 ng/mL) to test pathway convergence. (**F**) AQP4-IgG and CHI3L1 each increase p65 phosphorylation at 6 hours; combined treatment further augments p65 activation (immunoblot phospho/total p65; *n* = 3). (**G**) Immunofluorescence at 24 hours shows increased nuclear p65, GFAP upregulation, and AQP4 internalization in response to AQP4-IgG; these effects are enhanced by CHI3L1 cotreatment. Scale bars: 20 μm; *n* = 5 slides (6 fields per slide). (**H**) qPCR confirms amplification of NF-κB–regulated cytokine mRNAs by CHI3L1 in the AQP4-IgG condition; heatmap normalized to Ctrl-IgG + vehicle (*n* = 3). (**I**) ELISA detects higher secreted TNF-α, IL-1β, IL-6, and C3 with AQP4-IgG + CHI3L1 versus either alone (24 hours; *n* = 3). Statistics: Data are mean ± SEM. Bar graph comparisons used 1-way ANOVA with Tukey’s post hoc test or Welch’s ANOVA with Dunnett’s T3 test for unequal variances. Non-significant comparisons are not shown. *P < 0.05; **P < 0.01; ***P < 0.001; ****P < 0.0001.

Prior work shows that CHI3L1 activates diverse pathways (MAPK/ERK, AKT/PKB, NF-κB, GSK-3β, Wnt/β-catenin) via receptor binding (3, 27–29). To define astrocyte-specific signaling, we stimulated primary astrocytes with graded CHI3L1 (Figure 3A). CHI3L1 robustly increased NF-κB (p65) phosphorylation in a dose-dependent manner, with little or no activation of MAPK/ERK, AKT/PKB, or Wnt/β-catenin (Figure 3B). Given the established role of NF-κB in neurotoxic astrocyte states (4, 30), these data implicate NF-κB as the principal CHI3L1 effector in astrocytes.

Focusing on NF-κB targets, we quantified 7 proinflammatory mediators (TNF-α, IL-1β, IL-6, IL-1α, CCL5, CCL7, and C3). CHI3L1 increased their transcripts (Figure 3C) and secretion (Figure 3D) in a dose-dependent fashion, indicating direct astrocyte activation. Thus, CHI3L1 drives an NF-κB–dependent, neurotoxic astrocyte program that amplifies cytokine release and may fuel NMO progression.

To characterize CHI3L1-mediated NF-κB pathway activation in the context of astrocyte activation initiated by AQP4-IgG — as occurs in NMO — we considered that the binding of anti-AQP4 IgG to surface AQP4 proteins on astrocytes leads to internalization and degradation of AQP4, mirroring events in NMO brains. Importantly, this binding induces CHI3L1 expression and provokes inflammatory features (1, 15, 17, 18, 21). To corroborate the induction of a reactive program in astrocytes by AQP4-IgG, we reanalyzed the astroglial transcriptome dataset from Walker-Caulfield et al. (17). Among the differentially expressed genes reported, we identified that a significant number are well-known direct targets of NF-κB–mediated transcription (Supplemental Figure 5A), suggesting elevated transcriptional activity of NF-κB in response to AQP4-IgG. To test whether this induction of NF-κB–mediated transcription is affected by secreted CHI3L1, we coadministered CHI3L1 or vehicle control alongside treatments of Ctrl-IgG or AQP4-IgG on primary astrocytes (Figure 3E). Both AQP4-IgG and CHI3L1 alone induced p65 phosphorylation and activated NF-κB, and the combination of AQP4-IgG and CHI3L1 further enhanced this induction (Figure 3F).

To further corroborate the effect of CHI3L1 on NF-κB transcriptional activities, we analyzed the nuclear translocation of p65 in astrocytes treated with Ctrl-IgG or AQP4-IgG, plus vehicle control or CHI3L1. We observed increased nuclear translocation of p65 caused by either AQP4-IgG or CHI3L1 alone, with the combination resulting in an even higher level of p65 nuclear translocation (Figure 3G). This suggests a synergistic effect where CHI3L1 amplifies the NF-κB signaling initiated by AQP4-IgG. To validate the effect of CHI3L1 on NF-κB–mediated transcription, we examined mRNA transcript levels of NF-κB target genes — a panel of selected proinflammatory cytokines (Figure 3H) — and their secretion from the treated astrocytes (Figure 3I). Consistently, coadministration of CHI3L1 enhanced the effects of AQP4-IgG, leading to greater expression and secretion of proinflammatory cytokines.

Together, these data indicate that CHI3L1 acts autocrinely on astrocytes during AQP4-IgG–driven activation and that NF-κB is the key downstream effector. This positions NF-κB as a central mediator of astrocyte activation and cytokine release in NMO. Accordingly, pinpointing the astrocytic CHI3L1 receptor that triggers NF-κB is a priority, both to clarify mechanism and to expose new therapeutic targets for interrupting this pathogenic cascade.

### CHI3L1 engages the astrocyte receptor RAGE to activate NF-κB signaling, mediating inflammatory toxicity and motor dysfunction in NMO.

**Figure 4:**
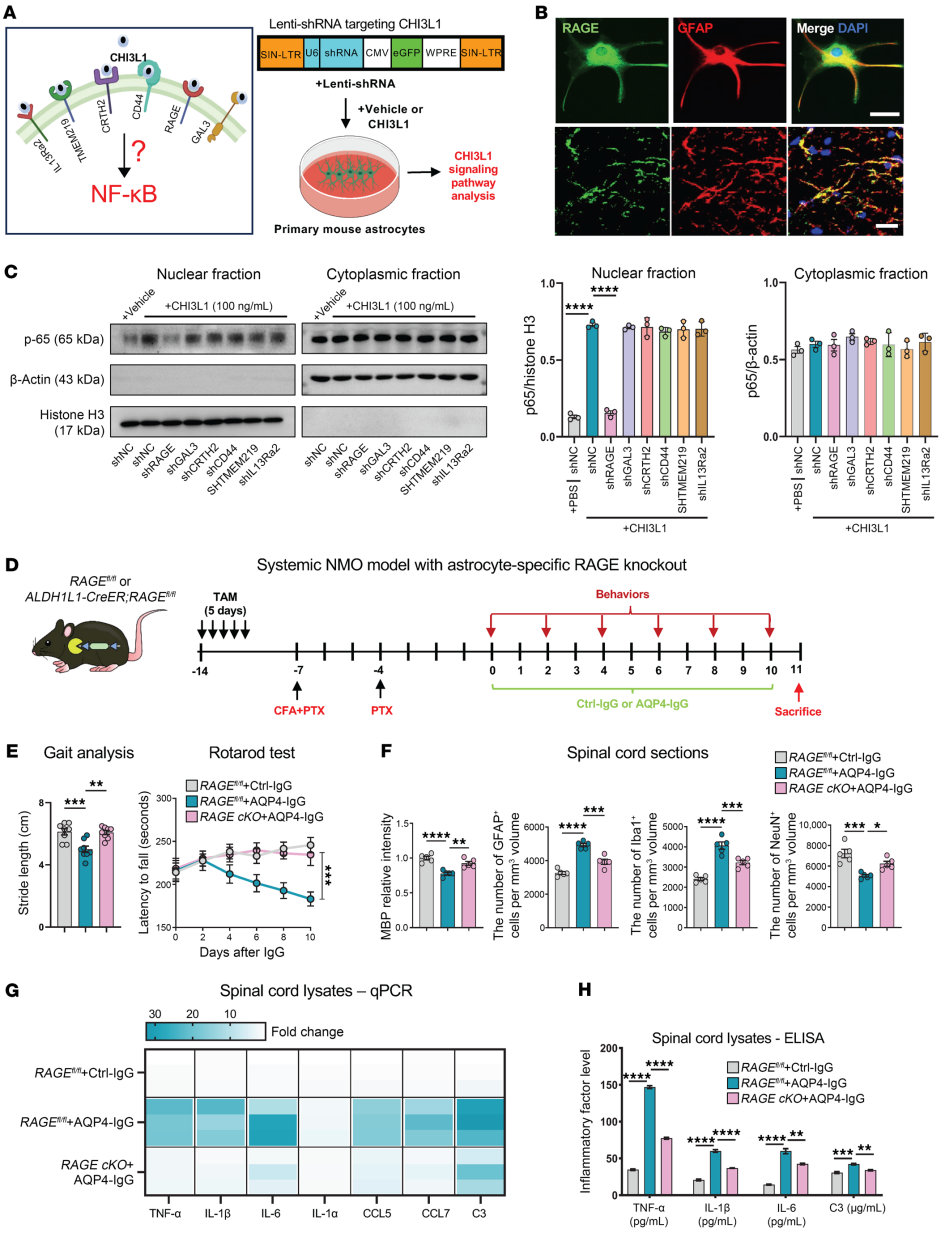
Astrocyte-specific RAGE deletion attenuates NMO pathology by blocking CHI3L1-driven NF-κB activation. (**A**) Candidate CHI3L1 receptors on astrocytes (IL-13Rα2, TMEM219, Gal-3, CD44, CRTH2, RAGE) and shRNA screen design: primary astrocytes transduced with lentivirus encoding GFP plus non-targeting shRNA (shNC) or receptor-targeting shRNA (>90% transduction). (**B**) RAGE is expressed in spinal astrocytes: colocalization of RAGE with GFAP in L4 sections from control mice. Scale bar: 20 μm. (**C**) Receptor knockdown screen identifies RAGE as required for CHI3L1-induced NF-κB: astrocytes expressing shNC or receptor shRNAs were treated with or without CHI3L1 (100 ng/mL, 6 hours); nuclear/cytoplasmic p65 quantified by immunoblot (histone H3 and β-actin as fraction controls) (*n* = 3). (**D**) Conditional astrocyte-specific RAGE knockout (RAGE cKO): *RAGE^fl/fl^* × ALDH1L1-CreERT2 with tamoxifen induction, followed by systemic NMO paradigm (AQP4-IgG or Ctrl-IgG), behavioral testing, and spinal histopathology. (**E**) RAGE cKO improves motor function: gait (stride length) and rotarod latency in *RAGE^fl/fl^* + Ctrl-IgG, *RAGE^fl/fl^* + AQP4-IgG, and RAGE cKO + AQP4-IgG groups (*n* = 8 per group). (**F**) RAGE cKO reduces demyelination and glial activation and preserves neurons: MBP intensity, GFAP^+^ and Iba1^+^ cell densities, and NeuN^+^ counts in L4 sections (*n* = 5 per group; 3 sections per mouse). (**G**) Proinflammatory transcript suppression in RAGE cKO: qPCR heatmap for NF-κB targets (Tnf, Il1b, Il6, Il1a, Ccl5, Ccl7, C3) in lumbar cord, normalized to *RAGE^fl/fl^* + Ctrl-IgG (*n* = 3 per group). (**H**) Reduced cytokine proteins in RAGE cKO: ELISA for TNF-α, IL-1β, IL-6, and C3 in lumbar cord lysates (*n* = 3 per group). *P < 0.05; **P < 0.01; ***P < 0.001; ****P < 0.0001.

CHI3L1 has been documented to activate intracellular signaling cascades for diverse cellular functions by engaging a well-characterized set of surface receptors, including interleukin-13 receptor subunit α2 (IL-13Rα2), transmembrane protein 219 (TMEM219), galectin-3 (Gal-3), CD44, chemoattractant receptor–homologous molecule expressed on Th2 cells (CRTH2), and the receptor for advanced glycation end products (RAGE) (4, 13, 27). Our prior study demonstrated that CHI3L1 adversely affects the proliferation and differentiation of neural stem cells and impairs hippocampal neurogenesis by interacting with CRTH2. In the current study, we aim to identify the specific receptor responsible for NF-κB activation by CHI3L1 in astrocytes (Figure 4A).

We assessed the expression of all known CHI3L1 receptors in astrocytes within the lumbar spinal cord and found that each is expressed (Figure 4B and Supplemental Figure 6A). To pinpoint the receptor predominantly responsible for CHI3L1-induced NF-κB activation, we introduced validated short hairpin RNAs (shRNAs) targeting these individual receptors into primary astrocytes via lentiviral transduction, using a scrambled non-targeting control shRNA (shNC) as a control (Figure 4A). By assaying p65 phosphorylation following CHI3L1 treatment, we discovered that depletion of RAGE — but not any of the other tested receptors — significantly dampened NF-κB activation (Figure 4C). This finding suggests that RAGE is the key CHI3L1 receptor mediating NF-κB activation in astrocytes during AQP4-IgG–induced astrocyte activation.

To further corroborate the role of RAGE, we employed an anti-RAGE antibody to antagonize ligand activation (31, 32) and used FPS-ZM1, a BBB-permeable, high-affinity inhibitor of RAGE (33–35). In primary astrocytes treated with CHI3L1, both the anti-RAGE antibody and FPS-ZM1 blocked the induction of p65 nuclear localization caused by CHI3L1 (Supplemental Figure 6B) and lessened the transcription and secretion of NF-κB target proinflammatory factors (Supplemental Figure 6, C and D). These results strengthen the evidence that RAGE mediates CHI3L1-induced NF-κB activation, thereby controlling astrocyte activation in NMO. Notably, RAGE has been strongly linked to Alzheimer’s disease and multiple neurodegenerative conditions (36, 37) and is currently the only known CHI3L1 receptor that activates NF-κB (38–40).

RAGE is a multi-ligand cell surface receptor belonging to the immunoglobulin superfamily. In addition to binding CHI3L1, it interacts with ligands such as advanced glycation end products (AGEs), S100/calgranulin proteins, high-mobility group box 1 protein (HMGB1), and amyloid-β peptide (4) (Supplemental Figure 6E). Importantly, soluble RAGE (sRAGE), a circulating form produced through alternative splicing or proteolytic cleavage, acts as a decoy receptor to sequester RAGE ligands, preventing their engagement with the full-length transmembrane RAGE (41). Studies have shown that decreased levels of sRAGE in patients with autoimmune disorders, such as MS (42), systemic lupus erythematosus (43), and Guillain-Barré syndrome (44), correlate with increased inflammatory activity and clinical manifestations, suggesting that reduced sRAGE may lead to enhanced RAGE activation and contribute to disease progression (41).

We first decided to directly target RAGE expression through a transgenic approach to delete RAGE specifically in astrocytes. We generated mice with inducible astrocyte-specific RAGE knockout (RAGE cKO) by breeding *RAGE^fl/fl^* mice with ALDH1L1-CreERT2 transgenic driver mice, enabling gene deletion upon tamoxifen administration. Employing the systemic NMO model with behavioral, histopathological, and biochemical analyses (Figure 4D), we found that ablation of RAGE in astrocytes (Supplemental Figure 6, F and G) largely abolished the pathological effects of AQP4-IgG–induced astrocyte activation. This was evidenced by improvements in motor function assessed by behavioral assays (Figure 4E). Histopathological examinations revealed that astrocyte-specific deletion of RAGE rescued demyelination and reduced activation of astrocytes and microglia within the lumbar spinal cord (Figure 4F). Biochemical assays on lumbar spinal cord tissues showed a reduction in proinflammatory activities in systemic NMO mice with astrocyte-specific deletion of RAGE (Figure 4, G and H).

Given our findings identifying RAGE as the major CHI3L1 receptor for astrocyte activation, we sought to determine whether clinical data support a role for RAGE in NMO. We analyzed a cohort of NMO patients (a total of 60, 4 male and 56 female) that we recently established, recruiting individuals with new-onset, AQP4-IgG–seropositive NMO during acute attacks who had not received steroids or immunomodulatory therapy, along with age-matched control subjects (Table 1). As with many autoimmune diseases, NMO predominantly affects females, who account for up to 90% of the patient population (45). Consistent with previous studies, CHI3L1 levels in cerebrospinal fluid (CSF) and serum were significantly elevated compared with those of controls (15), and the increase in CHI3L1 levels correlated with the severity of neurological deficits (Supplemental Figure 7A). We also measured serum levels of sRAGE using established ELISA methods and observed a moderate yet significant reduction in NMO patients. Importantly, the reduction in sRAGE serum levels correlated with the severity of neurological deficits (Supplemental Figure 7B), suggesting enhanced RAGE activation in NMO and highlighting the potential of sRAGE as a marker for the autoimmune-mediated inflammatory activity and clinical outcomes in NMO.

To assess whether supplementation with sRAGE or administration of FPS-ZM1 could alleviate NMO-relevant pathology and motor impairments, we applied these treatments in the systemic NMO mouse model (Supplemental Figure 7C). Both sRAGE and FPS-ZM1 improved motor function in systemic NMO mice (Supplemental Figure 7D). Confocal microscopy examinations of lumbar spinal cord sections revealed that sRAGE and FPS-ZM1 administration ameliorated the loss of ventral horn neurons, restored myelination, and reduced activation of astrocytes and microglia within the lumbar spinal cord of NMO mice (Supplemental Figure 7E). Further biochemical analyses of the lumbar spinal cord tissues showed that both treatments reduced the expression and secretion of proinflammatory factors (Supplemental Figure 7, F and G). Our results from these two independent pharmacological approaches to block RAGE function strongly support the involvement of RAGE in NMO pathogenesis initiated by AQP4 antibodies.

Together, these data show that CHI3L1 engages RAGE to activate NF-κB in astrocytes, driving inflammatory neurotoxicity in NMO. Blocking this axis — pharmacologically or via astrocyte-specific RAGE deletion — attenuated demyelination, glial activation, and motor deficits, highlighting the CHI3L1-RAGE interaction as a promising therapeutic target and clarifying its role in NMO pathogenesis.

### STAT3 activation in astrocytes induces CHI3L1 expression, driving demyelination in NMO.

Our work has revealed how astrocyte-secreted CHI3L1 drives proinflammatory signaling and neurotoxicity, including demyelination and motor deficits. What remain unclear are the upstream triggers of CHI3L1 induction in astrocytes — particularly after AQP4-IgG attack — knowledge that is essential for fully understanding NMO pathogenesis and informing targeted therapies.

**Figure 5:**
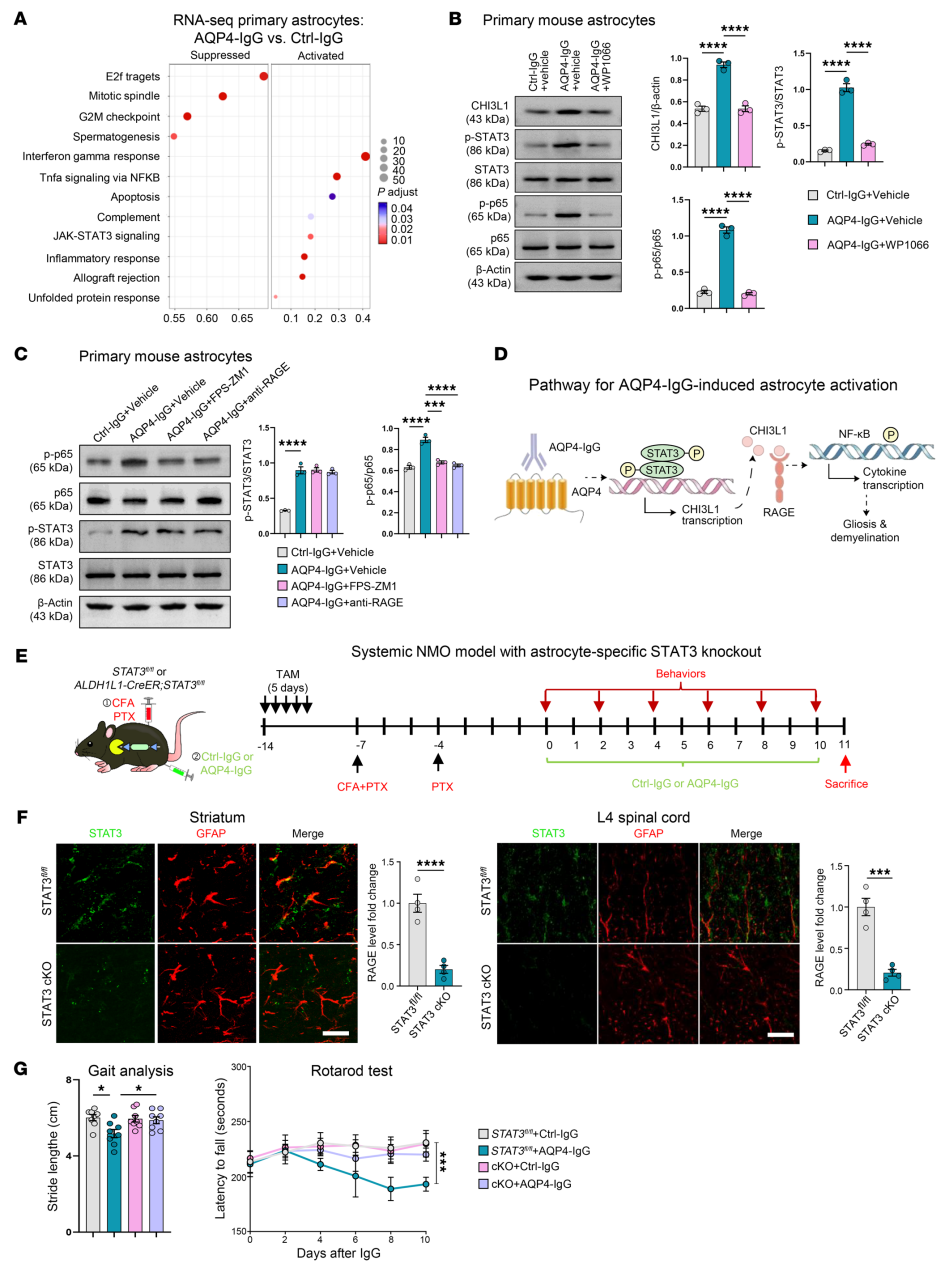
Astrocytic STAT3 drives CHI3L1 induction and NF-κB activation to mediate NMO pathology. (**A**) KEGG pathway analysis of differentially expressed genes from primary astrocytes treated with AQP4-IgG versus Ctrl-IgG identifies JAK/STAT3 among the top pathways activated by AQP4 autoantibody exposure. (**B**) AQP4-IgG activates STAT3 and NF-κB and induces CHI3L1 in primary astrocytes; WP1066 (STAT3 inhibitor) suppresses p-STAT3, p-p65, and CHI3L1 levels. Immunoblot densitometry shown as CHI3L1/β-actin and phospho/total ratios for STAT3 and p65 (*n* = 3 experiments). (**C**) Cultures were treated with AQP4-IgG or Ctrl-IgG in the presence of FPS-ZM1 or sRAGE; no exogenous CHI3L1 was added. RAGE blockade (FPS-ZM1 or sRAGE) does not affect AQP4-IgG–evoked p-STAT3 but reduces p-p65, placing STAT3 upstream of CHI3L1/RAGE/NF-κB. Phospho/total immunoblot ratios are shown (*n* = 3 per group). (**D**) Model: AQP4-IgG triggers astrocytic STAT3 activation → CHI3L1 expression/secretion → autocrine engagement of RAGE → NF-κB–dependent proinflammatory signaling. (**E**) Conditional astrocyte-specific STAT3 knockout (STAT3 cKO): *STAT3^fl/fl^* × ALDH1L1-CreERT2 with tamoxifen induction, followed by systemic NMO paradigm (AQP4-IgG or Ctrl-IgG), behavioral testing, and spinal histopathology. (**F**) Efficiency of STAT3 depletion in astrocytes confirmed in striatum and L4 spinal cord by IHC (~80% reduction in STAT3 signal in GFAP^+^ cells) in STAT3-cKO versus *STAT3^fl/fl^* controls. Scale bar: 20 μm. (**G**) STAT3 cKO improves motor outcomes: gait (stride length) and rotarod latency across *STAT3^fl/fl^* + Ctrl-IgG, *STAT3^fl/fl^* + AQP4-IgG, STAT3 cKO + Ctrl-IgG, and STAT3 cKO + AQP4-IgG groups (*n* = 8 per group). Statistics: Data are mean ± SEM. Bar graph comparisons in **B**, **C**, and **F** used 1-way ANOVA with Tukey’s post hoc test or Welch’s ANOVA with Dunnett’s T3 test for unequal variances. Rotarod latency was analyzed by 2-way ANOVA (**G**). Non-significant comparisons are not shown. *P < 0.05; ***P < 0.001; ****P < 0.0001.

Prior studies have demonstrated multiple signaling mechanisms that directly stimulate CHI3L1 transcription in peripheral immune cells (4). To determine the upstream mechanisms promoting CHI3L1 expression in astrocytes, we reanalyzed our transcriptomic dataset of primary astrocytes treated with AQP4-IgG, focusing on potential pathways that induce CHI3L1 expression as an early step of astrocyte activation. We performed Kyoto Encyclopedia of Genes and Genomes (KEGG) pathway enrichment analyses and found that the JAK/STAT3 signaling pathway was significantly activated in astrocytes treated with AQP4-IgG compared with vehicle control (Figure 5A). Notably, STAT3 is a transcription factor abundantly expressed in astrocytes and is well known to initiate the transcription of key genes involved in immune and inflammatory activities (46, 47). This finding was further confirmed by independent gene set enrichment analysis (GSEA; Supplemental Figure 8A). Moreover, by focusing on the common differentially expressed genes identified by our group and an independent study, we uncovered a significant enrichment of genes associated with the JAK/STAT3 signaling pathway (Supplemental Figure 8B). These bioinformatic approaches support the notion that JAK/STAT3 is a promising candidate for mediating the effects of AQP4 autoantibody attack, conveying the astrocyte activation signal to induce CHI3L1 expression and downstream activation of the RAGE receptor and NF-κB pathway.

In cultures of primary mouse astrocytes, we confirmed that AQP4-IgG treatment, compared with Ctrl-IgG treatment, induced phosphorylation of STAT3, along with phosphorylation of p65 and increased CHI3L1 expression (Figure 5B). To test the direct involvement of STAT3 pathway activation in the NF-κB pathway downstream of CHI3L1 action, we used WP1066, a selective inhibitor of STAT3 phosphorylation currently used in clinical trials for pediatric and adult brain tumors (48). Coadministration of WP1066 significantly suppressed the effects of AQP4-IgG treatment, including the phosphorylation of both STAT3 and NF-κB signaling components, and inhibited CHI3L1 expression (Figure 5B). This finding suggests a key role for STAT3 in astrocyte activation by AQP4-IgG, acting upstream of CHI3L1 expression and subsequent engagement of the RAGE receptor to activate the NF-κB pathway.

While STAT3, p65, and RAGE are major modulators or effectors in the AQP4-IgG–induced effects, their inter-pathway interactions can be complex. To further dissect the specific molecular mechanisms, we employed the anti-RAGE antibody to antagonize ligand activation (31, 32) and also used the selective RAGE inhibitor FPS-ZM1 (33–35) (Supplemental Figure 6B). In primary astrocytes treated with AQP4-IgG, we observed that blocking RAGE inhibited p65 phosphorylation; however, treatment with anti-RAGE or FPS-ZM1 did not affect STAT3 phosphorylation (Figure 5C). These findings elucidate the role of STAT3 in mediating the AQP4-IgG effect on astrocyte activation by inducing CHI3L1 expression, which then activates the RAGE receptor to trigger NF-κB pathway activation (Figure 5D). This sequential activation highlights the hierarchical relationship between these signaling molecules in astrocyte activation.

Guided by our in vitro finding that AQP4-IgG activates STAT3 in astrocytes, we combined a STAT3-floxed strain with an adeno-associated virus (AAV) strategy to generate an inducible, astrocyte-specific knockout in the focal NMO model. Specifically, the STAT3-floxed mice (*STAT3^fl/fl^*) received striatal injections of Ctrl-IgG or AQP4-IgG, along with AAVs expressing GFP-tagged Cre recombinase (AAV-Cre) or GFP alone (AAV-GFP) driven by the astrocyte-specific GfaABC1D promoter (Supplemental Figure 9A). One week later, GFP largely colocalized with glial fibrillary acidic protein (GFAP), confirming astrocyte-restricted transduction (Supplemental Figure 9B). In unfixed striata, immunoblotting showed greater than 70% STAT3 reduction in AAV-Cre versus AAV-GFP controls (Supplemental Figure 9C). In this focal NMO setting, astrocytic STAT3 loss dampened astrogliosis and microgliosis and, importantly, rescued myelin loss (Supplemental Figure 9D), indicating that astrocyte STAT3 is a key driver of AQP4-IgG–induced demyelinating inflammation.

To address the potential for incomplete deletion with the focal AAV strategy and to test functional outcomes, we next used a systemic, inducible genetic approach. Mice with conditional astrocyte-specific STAT3 knockout (STAT3 cKO; *STAT3^fl/fl^* × ALDH1L1-CreERT2) received tamoxifen induction, followed by the systemic NMO paradigm (AQP4-IgG or Ctrl-IgG), behavioral testing, and spinal cord histopathology (Figure 5E). Immunohistochemistry confirmed efficient astrocyte-restricted deletion (~80% reduction of STAT3 signal in GFAP^+^ cells) in both striatum and L4 spinal cord of STAT3-cKO mice compared with *STAT3^fl/fl^* controls (Figure 5F), and such STAT3 deletion significantly reduced the activation of astrocytes and microglia (Supplemental Figure 9E). Functionally, STAT3 ablation improved motor performance — normalizing stride length and prolonging rotarod latency — across the 4 experimental groups (*STAT3^fl/fl^* + Ctrl-IgG, *STAT3^fl/fl^* + AQP4-IgG, STAT3 cKO + Ctrl-IgG, STAT3 cKO + AQP4-IgG) (Figure 5G). Together, the focal and systemic loss-of-function studies converge to show that astrocytic STAT3 is a critical upstream driver of AQP4-IgG–evoked gliosis and demyelination.

Taken together, these results show that astrocytic STAT3 inhibition lowers CHI3L1, lessens NMO-like lesions, and improves motor function. Because STAT3 lies upstream of CHI3L1 and NF-κB, it represents a compelling therapeutic entry point for primary astrocytopathies such as NMO.

### STAT3 inhibition by WP1066 ameliorates demyelination and motor dysfunction in NMO models.

**Figure 6:**
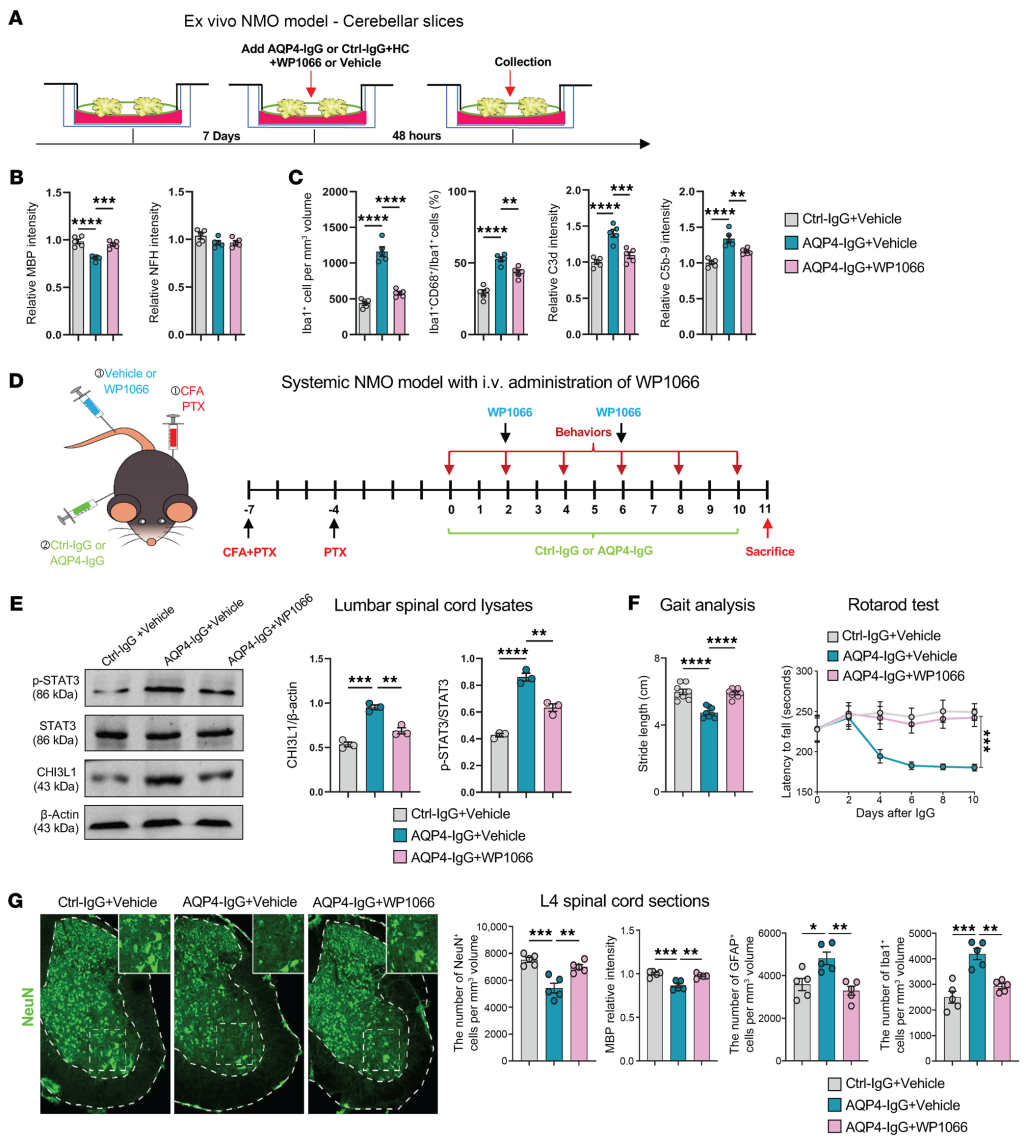
STAT3 inhibition with WP1066 ameliorates demyelination and motor deficits in NMO models. (**A**) Ex vivo paradigm: organotypic cerebellar slices treated 48 hours with AQP4-IgG or Ctrl-IgG with or without WP1066 (100 ng/mL), followed by immunostaining for myelination and glial activation. (**B**) WP1066 reduces AQP4-IgG–induced demyelination; MBP fluorescence normalized to Ctrl-IgG + vehicle = 1.0. NFH marks axonal process integrity. *n* = 5 slices per group (3 sections per slice). (**C**) WP1066 attenuates microgliosis and complement activation; Iba1^+^ total and Iba1^+^CD68^+^ activated microglia densities, C3d levels, and MAC (C5b-9) quantified relative to Ctrl-IgG + vehicle. *n* = 5 slices per group. (**D**) In vivo design: systemic NMO model with WP1066 (10 μg/mouse) or vehicle. Groups: Ctrl-IgG + vehicle, AQP4-IgG + vehicle, AQP4-IgG + WP1066. (**E**) WP1066 suppresses STAT3 activation and CHI3L1 induction in lumbar spinal cord; immunoblot densitometry shown as p-STAT3/STAT3 and CHI3L1/β-actin (*n* = 3). (**F**) WP1066 improves motor function in systemic NMO mice; gait (stride length) and rotarod latency (*n* = 8 per group). (**G**) WP1066 mitigates spinal demyelination and glial activation and preserves neurons; MBP intensity, GFAP^+^Iba1^+^ cell densities, and NeuN^+^ counts in L4 sections. Scale bars: 100 μm; *n* = 5 mice per group (3 sections per mouse). Statistics: Mean ± SEM. Bar graphs analyzed by 1-way ANOVA with Tukey’s post hoc test; rotarod latency by 2-way ANOVA (**F**). Non-significant comparisons are not shown. *P < 0.05; **P < 0.01; ***P < 0.001; ****P < 0.0001.

To examine whether STAT3 inhibition could serve as a promising therapeutic strategy for addressing demyelination and motor dysfunction in NMO, we used the STAT3 inhibitor WP1066. We first tested WP1066 in our ex vivo NMO model based on cerebellar slice cultures (Figure 6A). Coadministration of WP1066 with AQP4-IgG significantly lessened the loss of myelin in the cultured cerebellar slices compared with treatment with AQP4-IgG alone (Figure 6B). Moreover, WP1066 markedly reduced the activation of astrocytes and microglia and decreased complement deposition and membrane attack complex formation induced by AQP4-IgG (Figure 6C). These ex vivo data support the efficacy of WP1066 in reversing inflammatory demyelination pathology resulting from AQP4-IgG–induced astrocyte activation in NMO.

Encouraged by these results, we next sought to determine the translational potential of WP1066 in vivo using our systemic NMO mouse model. WP1066 was administered intravenously to young adult wild-type mice, alongside systemic administration of AQP4-IgG or Ctrl-IgG following chemical disruption of the blood-brain barrier and blood–spinal cord barrier. The treated mice underwent behavioral assessments, including gait analysis and the rotarod test, before being sacrificed for histopathological examination of lumbar spinal cord sections (Figure 6D).

To assess the in vivo effect of WP1066 on STAT3 activation, we collected spinal cord tissue lysates to analyze STAT3 phosphorylation and CHI3L1 protein levels. Consistent with our in vitro and ex vivo results, WP1066 treatment robustly inhibited STAT3 phosphorylation and reduced CHI3L1 induction in systemic NMO mice treated with AQP4-IgG, compared with those receiving Ctrl-IgG (Figure 6E). Behavioral assays demonstrated that WP1066 significantly improved motor function in systemic NMO mice, bringing performance close to the level of control mice. Specifically, gait analysis showed a significant increase in stride length, and the rotarod test revealed a prolonged latency before the mice fell from the rotating cylinder (Figure 6F).

Histopathological examinations of lumbar spinal cord sections revealed that WP1066 treatment rescued the loss of ventral horn neurons in NMO mice and significantly improved myelination. This was accompanied by reduced activation of astrocytes and microglia (Figure 6G). These findings indicate that WP1066 effectively mitigates both the demyelinating and the neurotoxic inflammatory aspects of NMO pathology in vivo.

Together, these data show that astrocytic STAT3 inhibition curbs AQP4-IgG–driven astrocyte activation, lowers CHI3L1, and blunts RAGE/NF-κB signaling — thereby reducing demyelination and improving motor function. STAT3 thus emerges as a powerful upstream therapeutic target for NMO.

## Discussion

Here, we define an astrocyte-intrinsic pathway contributing to NMO demyelination. AQP4 autoantibodies activate STAT3 in astrocytes, inducing CHI3L1, which engages RAGE autocrinely to activate NF-κB, amplify gliosis, and drive demyelination with motor deficits. Pharmacological blockade of STAT3, CHI3L1, or RAGE ameliorated pathology and improved outcomes in ex vivo and in vivo models. Thus, astrocytes act as active mediators in NMO and present tractable therapeutic targets.

Despite major advances in NMO immunopathology, most work has emphasized microglia as key mediators of autoimmune neuroinflammation (49, 50). Here we place astrocytes at the center of disease amplification. We show that astrocytes are not passive targets of AQP4-IgG but active drivers of inflammatory damage: CHI3L1 — best known as an intercellular inflammatory mediator — also acts autocrinely on astrocytes, engaging the RAGE→NF-κB pathway, heightening astrocyte activation, and promoting cytokine release. To our knowledge, this is the first demonstration that CHI3L1 directly fuels astrocyte activation in an autocrine loop, reframing NMO as an astrocyte-intrinsic amplifier of CNS inflammation and demyelination and opening tractable astrocyte-targeted therapeutic avenues.

White matter injury and demyelinating lesions are hallmarks of primary astrocytopathies — including Alexander disease (51), autoimmune GFAP astrocytopathy (52), and vanishing white matter (53), in addition to NMO. In Alexander disease and autoimmune GFAP astrocytopathy, astrocytic cytoskeletal disruption and maladaptive stress responses secondarily undermine oligodendrocyte survival and myelin maintenance, producing progressive leukoencephalopathy (51, 52). Recent work in vanishing white matter shows that astrocyte-selective eIF2B mutations precipitate myelin pathology in vivo, directly linking astrocyte dysfunction to oligodendrocyte failure (53). Across these settings, loss of trophic/metabolic support and astrocyte-derived cytokines/ROS create a hostile milieu that stresses oligodendrocytes and impairs myelination (54, 55). Our data add a mechanistic layer: CHI3L1-driven RAGE/NF-κB signaling switches astrocytes into a neurotoxic state that amplifies gliosis and compromises myelin. While the precise downstream events within oligodendrocytes remain to be mapped, targeting this upstream astrocyte program offers a principled route to forestall oligodendrocyte dysfunction and preserve myelin across astrocytopathies.

The significance of CHI3L1 in inflammatory CNS disorders is well established by us and others — extending beyond a biomarker to a mediator of pathogenesis (13, 15). Yet broad inhibition is challenging because CHI3L1 also supports homeostasis: global loss worsens stroke (56) and promotes tumor metastasis (57). Our data resolve this dilemma by defining an astrocyte-intrinsic pathway. Targeting the CHI3L1→RAGE→NF-κB axis within astrocytes suppresses maladaptive inflammation and demyelination in NMO while sparing beneficial CHI3L1 functions, offering a precise therapeutic entry point.

Limitations and future directions include the following. Although our datasets converge on STAT3 as the principal upstream driver of CHI3L1 in astrocytes, unbiased analyses also indicate an interferon-responsive signature after anti-AQP4 exposure (Figure 5A and Supplemental Figure 8B), consistent with interferon signaling reported in NMO spectrum disorder (NMOSD) samples (58, 59). We did not dissect STAT3-centric transcription from potential IFN–JAK/STAT crosstalk; planned studies will use interferon blockade or receptor-specific perturbations to test how interferon tone shapes CHI3L1 induction and downstream RAGE/NF-κB signaling. In addition, CHI3L1 is produced by microglia, macrophages, neutrophils, and lymphoid subsets and can act as a chemoattractant (60). Our passive-transfer and slice models underrepresent peripheral/adaptive immunity, so CHI3L1-directed therapies may show stage- or endotype-specific effects. We therefore view astrocytic CHI3L1 as an amplifier that can cooperate with immune cell–derived CHI3L1 in more inflammatory contexts. Testing astrocyte-specific CHI3L1 or RAGE deletion in active-immunization models — where astrocyte loss and leukocyte recruitment are stronger (23, 24) — would help distinguish necessity from amplification, recognizing these models’ variable penetrance and technical demands. As therapies advance, stratification by disease stage and immune milieu, and validation in models that better recapitulate systemic immunity, will be crucial for responsible translation.

Finally, the pharmacological tools used here — the STAT3 inhibitor WP1066 and the RAGE inhibitor FPS-ZM1 — are strong candidates for translation. Both have existing safety data and clinical testing in other indications, making them plausible for repurposing to NMO. By targeting discrete nodes in the astrocyte-intrinsic CHI3L1→RAGE→NF-κB axis, they offer a focused way to blunt pathogenic inflammation without broadly disrupting CHI3L1 physiology. Next steps include dose optimization, long-term efficacy/safety in NMO models, and progression toward early-phase clinical studies in NMO and related astrocytopathies. These findings lay a practical foundation for mechanism-guided therapies that address astrocyte-driven demyelination.

## Methods

### Sex as a biological variable.

Both sexes were included throughout. In the clinical cohort, male and female patients with NMO and matched controls provided serum and CSF; sex-stratified analyses were performed, and any dimorphic effects are reported. In animal studies, equal numbers of adult males and females were used. Pilot data showed no sex-dependent differences in baseline measures or responses to AQP4-IgG, CHI3L1 manipulation, or drugs, so the study was powered for overall effects. Because cellular, histological, and behavioral outcomes were parallel in both sexes, findings are expected to generalize across sexes.

### Clinical information and biological sample collection.

A total of 110 participants (Table 1) were enrolled at the Third Affiliated Hospital of Sun Yat-Sen University from 2019 to 2022: 60 NMO patients (4 male, 56 female) and 50 age-matched controls (24 male, 26 female). NMO was diagnosed per 2015 International Panel for NMO Diagnosis criteria (61) with confirmed AQP4-IgG seropositivity; only newly diagnosed, untreated patients were included. All NMO patients provided serum for CHI3L1 and sRAGE and underwent neurological evaluation (including EDSS); 30 also provided CSF for CHI3L1. Controls comprised 20 healthy volunteers (serum for CHI3L1/sRAGE and Ctrl-IgG purification; Supplemental Figure 1A) and 30 individuals with benign neurological symptoms who underwent CSF testing and were free of infection, inflammation, or structural CNS disease. Each participant contributed 1 serum and/or CSF sample.

### IgG purification.

Immunoglobulin G (IgG) was purified as previously described (15, 17, 18) from plasma of AQP4-IgG–seropositive NMO patients and healthy volunteers. Protein A beads (71149800-EG, GE) were used, with elution in 0.1 M glycine-HCl (pH 2.5). Eluates were concentrated (Amicon Ultra-15; 100 kDa), sterile-filtered (0.22 μm), and stored at –80°C. Samples were designated AQP4-IgG or Ctrl-IgG. All donors gave informed consent; protocols were approved by the Ethics Committee of the Third Affiliated Hospital of Sun Yat-Sen University.

### Primary astrocyte isolation and culture.

Primary astrocytes were prepared as previously described (15) from postnatal day 0 (P0) C57BL/6 mouse brains by enzymatic dissociation and cultured in DMEM/F12 with 10% FBS. After 9–10 days in vitro, mixed glia were shake-off–purified to deplete microglia/oligodendroglia, yielding more than 90% GFAP^+^ astrocytes. For assays, cells were exposed to Ctrl-IgG or AQP4-IgG (each 100 ng/mL) with or without recombinant CHI3L1 (100 ng/mL) for 24 hours. Astrocyte-conditioned medium was then collected after a further 24 hours in fresh medium, clarified (0.22 μm), and used immediately or stored at 4°C (<24 hours). Additional experimental details are provided in Supplemental Methods.

### Organotypic cerebellar slice culture models of NMO.

Organotypic cerebellar slices were prepared from P7 mouse cerebella and maintained at the air-liquid interface on porous inserts for 7 days before treatment, as described previously (16). On day 8, slices were exposed for 48 hours to Ctrl-IgG or AQP4-IgG with human complement, with or without modulators (CHI3L1, anti-CHI3L1, anti-RAGE, FPS-ZM1, sRAGE, or WP-1066; doses are given below). Full procedural details are provided in Supplemental Methods.

### Isolation of primary mouse OPCs and in vitro differentiation for myelination assays.

Primary oligodendrocyte precursor cells (OPCs) were isolated from P5–P8 mouse cortex by immunopanning and differentiated on poly-d-lysine/laminin substrates following Dugas and Emery (25), with minor modifications. Cells were expanded in PDGF-AA/NT-3–containing growth medium and switched to T3/CNTF/clemastine differentiation medium for maturation and myelination assays. Recombinant CHI3L1 (500 ng/mL) was applied at defined stages as indicated. Full details are provided in Supplemental Methods.

### Experimental animals.

Animals were housed on a 12-hour light/dark cycle with ad libitum food and water. Wild-type C57BL/6J female mice (6–8 weeks old) and Sprague-Dawley female rats (200–250 g) were from Vital River. Astrocyte-specific knockouts were generated as follows: Chil1 cKO (*Chil1^fl/fl^* × ALDH1L1-CreERT2), RAGE cKO (*RAGE^fl/fl^* × ALDH1L1-CreERT2), and STAT3 cKO produced either by AAV-Cre in *STAT3^fl/fl^* (focal model) or by *STAT3^fl/fl^* × ALDH1L1-CreERT2 (systemic model). Strains were ALDH1L1-CreERT2 (031008, The Jackson Laboratory), *STAT3^fl/fl^* (016923, The Jackson Laboratory), *RAGE^fl/fl^* (*Ager^fl/fl^*; NM-CKO-2116109, Shanghai Model Organisms), and *Chil1^fl/fl^* (RDDC/Cyagen; exons 3–5 floxed). Additional details, including PCR primer sequences for genotyping, are provided in Supplemental Methods.

### Tamoxifen administration to achieve astrocyte-specific gene deletion.

Tamoxifen (HY-13757A, MedChem Express) was prepared in corn oil at a concentration of 30 mg/mL and injected i.p. into mice at a dose of 180 mg/kg for 5 consecutive days.

### Systemic NMO mouse model.

We adapted a passive-transfer NMOSD paradigm (16) with transient barrier permeabilization. Mice received CFA/H37Ra s.c. on day –7 and pertussis toxin i.p. on days –7 and –4. From day 0, animals were injected daily i.p. for 10 days with AQP4-IgG or Ctrl-IgG (20 mg/mL, 200 μL). Cohorts received i.v. agents via the caudal vein on days 2 and 6: CHI3L1 (1 μg/mouse), anti-CHI3L1 (10 μg), FPS-ZM1 (10 μg), anti-RAGE (10 μg), sRAGE (10 μg), or WP1066 (10 μg). Additional procedural details are provided in Supplemental Methods.

### Motor function assessments.

Locomotor function was evaluated using 2 behavioral tests: the gait analysis and rotarod test, performed every other day from day 0 to day 10 after the administration of Ctrl-IgG or AQP4-IgG. In the rotarod test, each mouse was placed on a cylinder rotating at an initial speed of 10 rpm, gradually increasing to 40 rpm over a 5-minute period. For the gait analysis, mice were monitored as they walked on a narrow band, with their performance captured using the Catwalk Assisted Gait Analysis System (XR-FP101, SHXINRUAN Corp.). The median stride length of the hind limb was calculated from 5 consecutive steps. The rotarod test was repeated 3 times with 15-minute intervals for each mouse, and the average fall latency of the 3 tests was recorded.

### Electron microscopy and g-ratio for myelination status.

Electron microscopy assessed spinal cord myelin. After perfusion with glutaraldehyde, 1 mm^3^ blocks were postfixed (2.5% glutaraldehyde), osmicated (1% OsO_4_ in 0.1 M PBS, pH 7.4, 2 hours, room temperature), dehydrated (graded ethanol/acetone), and embedded (EMbed 812) (Servicebio). Transverse ultrathin sections (60–80 nm) were stained with uranyl acetate/lead citrate and imaged on an HT7800 transmission electron microscope (Hitachi). G-ratios (axon/fiber diameter) were quantified in ImageJ (NIH) to evaluate myelin thickness.

### Focal NMO mouse model with stereotaxic injections into striatum.

We induced focal NMOSD-like lesions by stereotaxic infusion into dorsal striatum as previously described (15), with minor adaptations. Under isoflurane anesthesia, mice received 6 μL per site of a premix containing AQP4-IgG or Ctrl-IgG (3 μL, 20 mg/mL), human complement (2 μL, 1 mg/mL), and 1 μL of anti-CHI3L1 (1 mg/mL), anti-RAGE (1 mg/mL), or PBS. Injections were delivered via a 26-gauge Hamilton needle over 5 minutes; the needle was left in place 10 minutes to minimize reflux, then withdrawn slowly. Wounds were closed and animals recovered on warming pads with routine postoperative monitoring. Full procedural details (coordinates, rates, and perioperative care) are provided in Supplemental Methods.

### Focal NMO rat model.

Rats were anesthetized with 2% isoflurane and placed in a stereotaxic frame. A 1 mm burr hole was drilled 3.5 mm lateral to bregma; a 26-gauge needle was lowered 5 mm. Over 15 minutes, 30 μL of mixture (24 μL NMO-IgG plus 6 μL CHI3L1 antibody at 1 mg/mL or PBS) was infused using a Legato 130 pump (KD Scientific). The needle was left in place for 10 minutes to prevent reflux, then withdrawn slowly. Incisions were sutured with absorbable thread, and animals were recovered on a warming pad under observation.

### 3.0-Tesla MRI.

Magnetic resonance imaging (MRI) scanning of rats was performed with a 3.0-tesla (3.0T) MRI scanner. T2-weighted images (T2WIs) were acquired as DICOM files with a thickness of 1 mm. The T2WIs were collected to analyze the lesion volume and borders by manually tracing each slice using ImageJ software.

### Immunofluorescence staining.

Mice were deeply anesthetized and transcardially perfused with PBS followed by 4% paraformaldehyde (PFA). Brains and spinal cords were postfixed overnight, cryoprotected in 30% sucrose, and coronally sectioned at 40 μm. Primary astrocytes and organotypic slices were fixed in 4% PFA at room temperature. Free-floating sections/cells were permeabilized/blocked in serum with Triton X-100, incubated with primary antibodies overnight (4°C), washed, and incubated with fluorophore-conjugated secondary antibodies, counterstained with DAPI, and imaged by confocal microscopy under identical acquisition settings. Each experiment was independently replicated at least 3 times. Detailed reagents and conditions are provided in Supplemental Methods.

### Quantitative real-time PCR.

mRNA was extracted from primary astrocytes and spinal cord homogenates using the miRNeasy kit (QIAGEN) according to the manufacturer’s instructions. The mRNA was then quantified and assessed for purity using a Nanodrop spectrophotometer (Thermo Fisher Scientific). cDNA was synthesized from 1 μg of mRNA using the SureScript First-Strand cDNA Synthesis Kit (Genecopoeia Co.). Quantitative real-time PCR analysis was performed using BlazeTaq SYBR Green qPCR Mix (Genecopoeia Co.) on an Applied Biosystems instrument (Thermo Fisher Scientific). Fold changes were determined using the ΔΔCt method, with GAPDH serving as the internal control. The specific sequences of primers used are detailed in Supplemental Methods.

### Immunoblotting.

Lysates from primary astrocytes or mouse spinal cord were prepared in RIPA buffer, resolved by SDS-PAGE (8%–12%), and transferred to PVDF. Membranes were blocked (5% milk), probed overnight (4°C) with primary antibodies, incubated with HRP-secondaries, and developed by chemiluminescence. Band intensities were quantified in ImageJ and normalized to β-actin (whole lysate) or histone H3 (nuclear fractions). Full details are provided in Supplemental Methods.

### Enzyme-linked immunosorbent assay.

Samples were collected from the mouse spinal cord and primary astrocytes, as well as from the serum and CSF of NMO patients or healthy controls. Serum was collected after centrifuging of blood at 7,200*g* for 15 minutes at 4°C. The levels of inflammatory factors were measured using commercial enzyme-linked immunosorbent assay (ELISA) kits according to the manufacturer’s instructions. The absorbance of each standard and sample was measured at 450 nm, and a standard concentration gradient was used to create a standard curve. The details of the ELISA kits are provided in Supplemental Methods.

### Statistics.

Numerical data are reported as mean ± SEM from at least 3 independent replicates. Normality was assessed by Shapiro-Wilk test with *P* > 0.05 indicating a normal distribution. Two-group comparisons used 2-tailed Student’s *t* tests; multiple groups used 1-way ANOVA with Tukey’s post hoc test. Rotarod latency and dendritic complexity were analyzed by 2-way ANOVA with Tukey’s post hoc test. Scatterplots display individual g-ratios and axon size distributions. Clinical data are descriptive; Expanded Disability Status Scale (EDSS) correlations with serum CHI3L1 and sRAGE used Pearson’s r. *P* values of less than 0.05 were considered significant. Analyses were performed in GraphPad Prism 10.

### Study approval.

Written informed consent was obtained from participants; the study was approved by the Ethics Committee of the Third Affiliated Hospital of Sun Yat-Sen University. All animal procedures complied with the NIH *Guide for the Care and Use of Laboratory Animals* (National Academies Press, 2011) and were approved by Brown University (IACUC 22-11-0003) and the Third Affiliated Hospital of Sun Yat-Sen University (SYSU-IACUC-2024-B0959).

### Data availability.

All data associated with this study are present in the paper or the Supplemental Methods. Values for all data points in graphs are reported in the Supporting Data Values file. Raw immunoblot data are reported in the full unedited blot and gel images file. The lentiviral constructs and the transgenic mouse line described in this study are available from the corresponding authors upon request with a Uniform Biological Material Transfer Agreement.

## Author contributions

HX, CT, WQ, and YAH conceptualized the study. WJ, LX, HL, YS, YL and XY developed methodology. HX, WJ, LX, FZ, PH, HL, and XY performed investigation. CT, HX, WJ, LX, FZ, and HL wrote the original draft of the manuscript. CT and YAH reviewed and edited the manuscript. CT, WQ, and YAH acquired funding. CT, WQ, and YWAH supervised the study. CT and YAH performed project administration.

## Funding support

This work is the result of NIH funding, in whole or in part, and is subject to the NIH Public Access Policy. Through acceptance of this federal funding, the NIH has been given a right to make the work publicly available in PubMed Central.

- National Natural Science Foundation of China grants 82471382 (to CT), 32100787 (to WQ), and 82401413 (to WJ).
- National Institutes of Health (NIH) grant R01AG083943 (to YAH).
- National Key R&D Program of China grants 2022ZD0214300 and 2022YFC3600600 (to CT and WQ).
- Science and Technology Plan Project of Guangzhou City Grants 202201020489 and 2023A04J1089 (to CT).
- Guangdong Basic and Applied Basic Research Foundation grant 2022B1515120042 (to WQ).
- Technology Innovation 2030–“Brain Science and Brain-Like Research” Major Project grant 2022ZD0208900 (to WQ).
- Blackman Family Fund for Multiple Sclerosis (to YAH).
- Taishan Scholars Program for Young Experts of Shandong Province tsqn202408349 (to WJ).
- Regional Innovation Development Joint Fund of the National Natural Science Foundation of China U24A20691 (to WQ).

## Supplementary Material

Supplemental data

Unedited blot and gel images

Supporting data values

## Figures and Tables

**Table 1:**
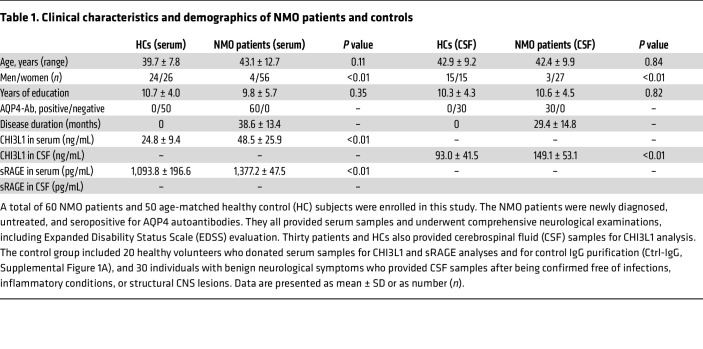
Clinical characteristics and demographics of NMO patients and controls

## Version 1 01/02/2026

Electronic publication
