# Supplementary material for: Astrocyte-intrinsic signaling of chitinase-like protein CHI3L1 drives inflammation and amplifies demyelination in neuromyelitis optica
Source: J Clin Invest. 2026 Jan 2;136(1):e195506. doi: 10.1172/JCI195506 (PMC12721897; doi:10.1172/JCI195506)
Supplement: Supplemental data [file jci-136-195506-s304.pdf]

Supplementary Materials

Methods:

Study design

In this study, we aimed to elucidate the astrocyte-intrinsic mechanisms contributing to the pathogenesis of neuromyelitis optica (NMO), with a particular focus on the CHI3L1–RAGE–NF-κB signaling axis. We employed a comprehensive experimental approach that included i*n vitro*, *ex vivo*, and in vivo models. Primary mouse astrocyte cultures were used to investigate the effects of AQP4-IgG on STAT3 activation and CHI3L1 expression, as well as downstream NF-κB signaling. Organotypic cerebellar slice cultures served as an *ex vivo* NMO model to assess demyelination and glial activation upon manipulation of CHI3L1 levels. For *in vivo* studies, we utilized both focal and systemic NMO mouse models, including conditional knockout mice with astrocyte-specific deletions of CHI3L1, STAT3, or RAGE. Pharmacological inhibitors such as WP1066 (a STAT3 inhibitor) and FPS-ZM1 (a RAGE antagonist) were administered to evaluate their therapeutic potential in mitigating NMO-related pathology. Behavioral assays, histopathological analyses, and molecular assessments were conducted to comprehensively evaluate the impact of targeting this signaling pathway on demyelination, neuroinflammation, and motor dysfunction. This multifaceted study design allowed us to dissect the roles of key signaling molecules within astrocytes and assess the translational potential of targeted therapies for NMO.

Primary astrocyte isolation and culture (expanded protocol)

Animals: C57BL/6J pups (P0) of either sex were used. All procedures followed institutional IACUC guidelines.

Reagents: DMEM (Gibco), DMEM/F12 (Gibco), FBS (PAN), penicillin–streptomycin (Gibco), 0.25% trypsin-EDTA (Gibco), non-essential amino acids (NEAA; MACKLIN), sodium pyruvate (Sigma), GlutaMAX or L-glutamine (Gibco), poly-D-lysine (PDL; 50 μg/mL), PBS (Ca^{2+}/Mg^{2+}-free). Antibodies: anti-GFAP (1:1000, CST #12389S). Stimuli: recombinant human CHI3L1 (Proteintech), purified AQP4-IgG or Ctrl-IgG (see main Methods), all endotoxin-tested (<0.1 EU/μg).

Dissection and dissociation: Whole brains from decapitated P0 pups were transferred to ice-cold DMEM containing 3% penicillin–streptomycin. Meninges were removed under a stereomicroscope. Tissue was minced and incubated with 0.25% trypsin-EDTA at 37 °C for 15 min with gentle trituration every 5 min. Enzymatic activity was quenched 1:1 with "N2A" stop medium (DMEM/F12 + 10% FBS + 1% PS). The suspension was passed through a 70 μm strainer, centrifuged 5 min at 1000 g, and the pellet was resuspended in astrocyte growth medium.

Astrocyte growth medium: DMEM/F12 supplemented with 10% FBS, 1% NEAA, 1 mM sodium pyruvate, 1% L-glutamine (or GlutaMAX), and 1% penicillin–streptomycin.

Plating: Flasks or plates were PDL-coated (50 μg mL^{-1}, 1 h, RT), rinsed in sterile water, and air-dried. Cells were seeded at ~2–3 × 10^5 cells cm^{-2} (e.g., 2–3 × 10^6 per T-25) and maintained at 37 °C, 5% CO_2. Medium was changed every 2–3 days for 9–10 days.

Shake-off purification (DIV9–10): Cultures were rinsed 3× with warm PBS to remove non-adherent cells, then shaken at 250 rpm, 37 °C for 20 h on an orbital shaker to detach microglia/oligodendroglia. Medium was replaced with fresh growth medium. Resulting monolayers were >90% $GFAP^+$ by immunocytochemistry; mycoplasma testing was negative.

Passaging (optional): For experiments, cells were used without passaging or after a single gentle detachment (Accutase) and replating onto PDL-coated plates at 60–80% confluence (e.g., 1.0–1.5 × 10^5 cells cm^{-2}) and allowed to recover overnight.

Astrocyte stimulations and ACM collection:

Treatments: Cells at 70–90% confluence were treated for 24 h with:

- Ctrl-IgG or AQP4-IgG, each at 100 ng mL^{-1}; and
- recombinant CHI3L1 (100 ng mL^{-1}) or vehicle.

  Stimuli were diluted in astrocyte growth medium. For time-course or dose-response experiments (where applicable), concentrations and durations are indicated in figure legends.

ACM preparation: After the initial 24 h treatment, cultures were rinsed once with warm PBS and replenished with fresh growth medium (no added IgG/CHI3L1) for an additional 24 h. Supernatants were collected, centrifuged 5 min at 300 g, filtered through a 0.22 µm PES or nylon filter, aliquoted, and used immediately or stored at 4 °C for <24 h (avoid freeze–thaw).

Quality controls: Parallel wells were fixed for GFAP immunostaining to confirm astrocyte purity, and cell viability was monitored (Trypan blue or live–dead assays). All treatments were performed in at least three independent cultures (biological replicates), each measured with technical duplicates/triplicates as appropriate.

Downstream assays: Cellular lysates were harvested for immunoblotting (e.g., p-STAT3/STAT3, p-p65/p65, CHI3L1) using RIPA buffer with protease/phosphatase inhibitors. RNA was extracted for qPCR of cytokines (e.g., Tnf, Il1b, Il6, Il1a, Ccl5, Ccl7, C3). For immunocytochemistry, cells were fixed in 4% PFA (10 min), blocked (5% normal donkey serum, 0.1% Triton X-100), and incubated with primary antibodies overnight at 4 °C.

Organotypic cerebellar slice culture models of NMO (detailed protocol)

Tissue preparation: P7 C57BL/6J pups of either sex were decapitated; cerebella were rapidly dissected into ice-cold HBSS (pH 7.2; Invitrogen). Transverse slices (350 µm) were cut with a McIlwain Tissue Chopper and transferred to 0.4 µm pore membrane inserts (Corning Transwell #3450) in 6-well plates at one slice per insert.

Culture medium and maintenance: Slices were cultured at the air–liquid interface (1.0 mL/well) in medium containing 50% MEM, 25% HBSS, 25% heat-inactivated horse serum, 2 mM L-glutamine, 200 mg/mL D-glucose, and 1% penicillin–streptomycin. Cultures were kept at 37 °C, 5% CO_2 with medium changes every 2–3 days for 7 days to allow recovery and myelination.

Experimental treatments (Day 8 → 48 h): Slices were treated in fresh medium with:

- IgG conditions: Ctrl-IgG or AQP4-IgG (as indicated in figure legends) plus human complement (1 mg mL^{-1}).
- Modulators: recombinant human CHI3L1 (500 ng mL^{-1}), anti-CHI3L1 antibody (100 ng mL^{-1}), anti-RAGE antibody (100 ng mL^{-1}), FPS-ZM1 (RAGE antagonist; 100 ng mL^{-1}), soluble RAGE (sRAGE; 100 ng mL^{-1}), or WP-1066 (STAT3 inhibitor; 100 ng mL^{-1}). Vehicle controls received PBS.

Endpoints: After 48 h, slices were fixed in 4% PFA and processed for confocal immunofluorescence (e.g., MBP for myelin, NFH for axons, GFAP for astrocytes, Iba1/CD68 for microglia, C3d and C5b-9 for complement activation). Imaging and quantification parameters are described in the figure legends; investigators were blinded to treatment during analysis.

Quality control and exclusions: Slices with mechanical damage or edge necrosis were excluded a priori. Each experiment included at least three biological replicates (independent slices from distinct litters), with technical duplicates where feasible.

Isolation of primary mouse OPCs and in-vitro differentiation for myelination assays

OPC isolation by immunopanning: Cortices from P5–P8 C57BL/6J pups (meninges removed) were papain-dissociated and gently triturated to a single-cell suspension. Sequential panning removed non-OPC lineages: (1) IgG/BSL-1 to deplete endothelial/meningeal cells, (2) anti-CD11b to remove microglia, and (3) anti-O4/GalC to exclude mature oligodendrocytes. OPCs were captured on A2B5 (or anti-PDGFRα) plates, released with trypsin, counted, and plated at $5–10\times10^4$ cells/cm^2 on poly-D-lysine/laminin–coated coverslips or plates.

Culture media and schedule

Growth (proliferation) medium: DMEM + 1% N2, 1% B-27, forskolin 10 µM, PDGF-AA 10 ng/mL, NT-3 1 ng/mL. Feed every 48 h; passage ≤2× to preserve OPC phenotype.

Differentiation/myelination medium: At ~60–70% confluence or day 2 post-plating, replace PDGF-AA with T3 (triiodothyronine) 40 ng/mL; add clemastine 1 µM and CNTF 10 ng/mL. Maintain 3–7 days for lineage marker maturation or 10–14 days for myelin-segment assays (on neurite or aligned nanofiber substrates). Refresh every 48 h.

CHI3L1 perturbations:

Recombinant CHI3L1 was added at 500 ng/mL at specified time points:

- Proliferation assays: during growth conditions.
- Differentiation assays: at induction (day 0) and/or 24 h later.
- Myelination assays: applied throughout the ensheathment period.

Readouts:

OPC identity and health were monitored by Olig2 and PDGFRα immunostaining. Proliferation was assessed by PCNA immunoblotting or EdU incorporation. Differentiation was quantified by MBP (and other stage markers as needed). Myelination on nanofibers/neurites was measured as MBP$^+$ ensheathment length/area. Analyses were performed blinded to condition.

Detailed Animal Methods

Regulatory approvals and housing:

Protocols were approved by Brown University IACUC (#22-11-0003) and Sun Yat-sen University IACUC (SYSU-IACUC-2024-B0959). Animals were maintained in SPF facilities (22 ± 2 °C, 40–60% humidity, 12-h light/dark cycle) with enrichment and ad libitum chow/water.

Strains and sources:

- ALDH1L1-CreERT2 (astrocyte-specific, tamoxifen-inducible): Jackson Laboratory, #031008.
- STAT3$^{flox/flox}$: Jackson Laboratory, #016923.
- RAGE$^{flox/flox}$: Shanghai Model Organisms, NM-CKO-2116109; Ager$^{flox/flox}$.
- Chil1$^{flox/flox}$: Rare Disease Data Center (RDDC)/Cyagen (CKOAIPL240812YY9); exons 3–5 floxed.
- Wild-type: C57BL/6J females (6–8 weeks) and Sprague–Dawley females (200–250 g), Vital River.

Breeding, genotyping, and randomization:

Breeders were maintained on a C57BL/6J background for ≥6 generations. Genotyping used vendor-recommended PCR primers; investigators were blinded to genotype during data acquisition/analysis. Littermates or age-matched controls were used when possible, with random assignment to treatment groups.

AAV-Cre focal knockout (STAT3$^{flox/flox}$, striatum):

For the focal STAT3 cKO, STAT3$^{flox/flox}$ mice received stereotaxic injections of AAV-GfaABC1D-Cre-GFP or AAV-GfaABC1D-GFP (control) into the striatum (AP +0.5 mm, ML ±2.0 mm, DV −3.0 mm; 0.8–1.0 μL/side; ~1–3×10^{12} vg/mL). Analyses were performed ≥7 days post-injection; astrocyte specificity was verified by GFP/GFAP co-localization (Fig. S9B), and STAT3 reduction confirmed by immunoblot (Fig. S9C).

Inclusion/exclusion, blinding, and sample size:

Animals with mistargeted injections, illness, or outlier weight (>2 SD from cohort mean) were prespecified for exclusion prior to unblinding. All imaging, behavioral testing, and histological quantification were performed **blinded** to genotype and treatment. Group sizes were informed by prior variance in similar assays and are reported in each figure legend.

Genotyping primers:

Chil1: forward: GCTACCCAACATGTCAATAGCTCA
Chil1: reverse: CATATGGTGGGCAATAATCTTGGA
RAGE: forward: CTCTGACAGAACGAGATGGGAGAT
RAGE: reverse: CCATAGAGCAAGAACCAGCACC
STAT3: forward: TTG ACC TGT GCT CCT ACAAAAA
STAT3: reverse: CCC TAG ATT AGG CCA GCA CA
ALDH1L1: CreERT2 Forward: CATATTGGCAGAACGAAAACGC
ALDH1L1: CreERT2 Reverse: CCTGTTTCACTATCCAGGTTACGG

Detailed Systemic NMO Protocol

Barrier permeabilization ("priming"):

- CFA/H37Ra: Complete Freund's Adjuvant containing heat-killed *M. tuberculosis* H37Ra (50 μg/site) was administered s.c. at four hindquarter sites on day −7 (total 200 μg/mouse).
- Pertussis toxin: 200 ng/mouse i.p. on days −7 and −4 to transiently increase BBB/BSCB permeability.

Antibody passive transfer:

- Reagents: Human AQP4-IgG or Ctrl-IgG purified from donor sera (see Fig. S1A; endotoxin <0.1 EU/mL).
- Dosing schedule: Day 0–9, i.p. once daily, 200 μL of IgG at 20 mg/mL.

Therapeutic interventions:

- Route/timing: Intravenous (caudal vein) on days 2 and 6 post-first IgG dose.
- Agents/doses (per mouse):
    - CHI3L1 recombinant protein, 1 μg.
    - Anti-CHI3L1 neutralizing mAb, 10 μg.
    - FPS-ZM1 (RAGE antagonist), 10 μg.
    - Anti-RAGE blocking mAb, 10 μg.
    - sRAGE, 10 μg.
    - WP1066 (STAT3 inhibitor), 10 μg.

- Vehicles matched by formulation; injection volume 100–150 μL.

Monitoring and endpoints:

- Behavior: Gait (stride length) and Rotarod (latency to fall) on days 10–12.
- Tissue collection: Lumbar spinal cord (L4) harvested 24–48 h after final behavioral testing for IHC/IF (AQP4, MBP, GFAP, Iba1, NeuN), qPCR (TNF-α, IL-1β, IL-6, IL-1α, CCL5, CCL7, C3), and ELISA (same cytokines).
- Blinding/randomization: Animals randomized to groups; assessors blinded to treatment. Pre-specified exclusions (e.g., injection failure, illness) applied prior to unblinding.

Detailed protocol for focal NMO model with striatal injections

Preparation and anesthesia:

- Induce/maintain with isoflurane (induction ~3–4%, maintenance ~1.5–2% in oxygen or air) via nose cone.
- Apply ophthalmic ointment; place the animal on a thermostated heating pad (37 °C).

Stereotaxic setup:

- Secure the head in a stereotaxic frame.
- Make a midline scalp incision; expose skull.
- Identify bregma and mark striatal target coordinates (dorsal striatum).
- Drill a ~1 mm burr hole at each target.

Infusate and delivery:

- Infusate (per site, total 6 μL):
  - 3 μL AQP4-IgG or Ctrl-IgG (final 20 mg/mL).
  - 2 μL human complement (1 mg/mL).
  - 1 μL of anti-CHI3L1 (1 mg/mL) or anti-RAGE (1 mg/mL) or PBS (vehicle).
- Load into a 26-gauge Hamilton syringe; lower to DV target slowly to avoid cortical compression.
- Infuse with a digital syringe pump (Legato 130) over 5 min (rate ~1.2 μL/min).
- Dwell 10 min post-infusion to reduce backflow; retract the needle in small increments.

Closure and recovery:

- Irrigate the site with sterile saline; close the scalp with absorbable sutures.
- Administer postoperative analgesia per IACUC protocol (e.g., buprenorphine).
- Recover on a warming pad; monitor until ambulatory, then daily for weight, grooming, and activity.

Quality control and exclusions:

- Exclude animals with evidence of mis-targeting, hemorrhage, or postoperative complications prior to unblinding.
- Randomization and blinded outcome assessment were used for histology and quantification.

Downstream analyses:

- Tissue collected at defined time points (e.g., 1–28 dpi for kinetics; 7 dpi for endpoint) for IHC/IF (AQP4, MBP, GFAP, Iba1), lesion mapping, and quantification as specified in figure legends.

Detailed immunofluorescence protocol

Tissue collection and sectioning:

- Euthanasia: IP pentobarbital, followed by PBS → 4% PFA perfusion.
- Post-fix: 4% PFA, 4 °C, overnight; cryoprotect in 30% sucrose until sunk.
- Sectioning: coronal 40 µm (brains; L4 spinal cord). Store free-floating in cryoprotectant (glycerol:ethylene glycol:0.1 M PB = 1:1:2) at 4 °C.

Cell and slice fixation:

- Primary astrocytes / cerebellar organotypic slices: 4% PFA, 1 h, RT → PBS ×3.

Permeabilization and blocking:

- Free-floating sections / astrocytes: 0.3% Triton X-100 in PBS with 10% normal goat or donkey serum, 1 h, RT.
- Organotypic slices: 0.5% Triton X-100, 30 min → 10% serum in PBS, 1 h, RT.

Primary antibody incubation (overnight, 4 °C):

The following primary antibodies were used: rabbit anti-AQP4 (1:1000, Santa Cruz Biotechnology, H-80, sc-20812), mouse anti-GFAP (1:500, Santa Cruz Biotechnology, sc-33673, clone 2E1, RRID: AB_627673), mouse anti-GFAP (1:500, CST, GA5, #3670), rabbit anti-MBP (1:500, CST, D8X4Q, 78896), mouse anti-NFH (1:500, CST, RMdO 20, 2836), rabbit anti-Iba1 (1:1000, Wako Chemicals, 019-19741), mouse anti-NeuN (1:500, Abcam, ab104224, clone 1B7), mouse anti-NF-κB-p65 (1:200, CST, L8F6, #6956), rabbit anti-CHI3L1 (1:500, Solarbio, K107483P), and rabbit anti-MAP2 (1:500, Abcam, ab32454).

Secondary antibody incubation (1 h, RT):

- Goat anti-rabbit Alexa 488(A-11008)/568(A-11011)/647(A-21245) (1:1000, Invitrogen)
- Goat anti-mouse Alexa 488(A-11029)/568(A-11004)/647(A-21236) (1:1000, Invitrogen)
- Goat anti-chicken Alexa 647(A-21449) (1:1000, Invitrogen)
- Wash PBS ×3 between steps; nuclei with DAPI (per manufacturer).

Imaging and replication:

- Confocal: Leica TCS SP8 (405/488/568/647 nm), 20×/40×/63×; single planes and z-stacks.
- Exposure/laser settings matched within an experiment; negative controls lacking primary included.
- Biological replication: ≥3 independent experiments (cells/slices) or n as specified (tissue sections).

Quantification and blinding:

- Regions of interest defined a priori (e.g., striatal lesion outline; L4 ventral horn).
- Background-subtracted mean fluorescence intensity and/or cell density (immunopositive cells per area) quantified in ImageJ/FIJI.
- Analysts blinded to group assignment; ≥3 sections per animal averaged to a single n.

Detailed immunoblotting protocol

Sample preparation:

- Tissue/cells: RIPA lysis (50 mM Tris-HCl pH 7.4, 150 mM NaCl, 1% NP-40, 0.5% sodium deoxycholate, 0.1% SDS) + protease/phosphatase inhibitors.
- Homogenize on ice, incubate 20–30 min, clear at 14,000 g, 15 min, 4 °C; collect supernatant.
- Determine protein concentration (BCA). For nuclear/cytosolic fractionation, use standard hypotonic buffer protocols; validate with Histone H3 (nuclear) and β-actin (cytosolic/whole).

SDS–PAGE and transfer:

- Mix with 4× Laemmli + DTT/β-ME; heat 95 °C, 5 min.

- Load 10–30 µg per lane on 8–12% gels; run at 120 V.
- Transfer to methanol-activated PVDF (0.2 µm) at 100 V, 60–90 min (wet) or per semi-dry settings.

Blocking and antibody incubation:

- Block 5% (w/v) non-fat milk in TBST, 1 h, RT.
- Primary antibodies (overnight, 4 °C, in TBST + 5% BSA for phospho-targets):
  The primary antibodies used were: mouse anti-NF-κB-p65 (1:1000, CST, clone L8F6, #6956), rabbit anti-p-NF-κB-p65 Ser536 (1:1000, CST, clone 93H1, #3033), mouse anti-β-actin (1:1000, Abcam, clone AC-15, ab6276), mouse anti-histone H3 (1:1000, Abcam, ab24834), mouse anti-STAT3 (1:1000, CST, #9139), rabbit anti-p-STAT3 (1:1000, CST, clone D3A7, #9145), and rabbit anti-CHI3L1 (1:1000, Solarbio, K109584P).
- Wash TBST ×3, 5 min each.
- HRP-conjugated secondary antibodies (goat anti-mouse/rabbit, 1:5000–1:10,000 in TBST), 1 h, RT; wash TBST ×3.

Detection and quantification:

- Develop with ECL (Millipore ECL Plus) and image on Tanon 5500 (or equivalent).
- Ensure exposures within linear range; avoid saturation.
- Quantify band densitometry in ImageJ/FIJI.
- Normalize phospho-signals to total protein (e.g., p-STAT3/STAT3, p-p65/p65); normalize total proteins to β-actin (whole lysate) or Histone H3 (nuclear).
- For each biological n, average technical replicates; report mean ± SEM and statistics as specified in the figure legends.

Quantitative real-time PCR (RT-qPCR) primer sequences (Forward/Reverse)

CCL5: CCTCACCATCCTCACTG/TCTTCTCTGGGTTGGCACAC
CCL7: ATCTCTGCCACGCTTCTGTG/CCTCTTGGGGATCTTTTGTTT
IL-1α: CAGTGAGACCTTCACTGAAG/CTGGAAGTCTGTCATAGAGG
C3: GCGCTTACCTGTAGGCATTGC/GCGCTTACCTGTAGGCATTGC
IL-1β: GGCTGCTTCCAAACCTTTGA/GAAGACACGGATTCCATGGT
TNF-a: GACTCAAATGGGCTTTCCGA/CCAGCCTCATTCTGAGACAGAG
IL-6: GCTTAATTACACATGTTCTCTGGGAAA/CAAGTGCATCATCGTTGTTCATAC
GAPDH: GGGTGTGAACCACGAGAAAT/ACTGTGGTCATGAGCCCTTC

Enzyme-Linked Immunosorbent Assay (ELISA) information (all from ZCIBIO Technology Co., Ltd)

Mouse IL-1β, ZC-37974; mouse TNF-α, ZC-39024; mouse IL-6, ZC-37988; mouse C3, ZC-38022; mouse sRAGE, ZC-38428; human CHI3L1, ZC-33434; human sRAGE, ZC-33908.

Figures and Legends

Figure S1. Preparation of human IgG and characterization of the focal AQP4-IgG model (related to Fig. 1)

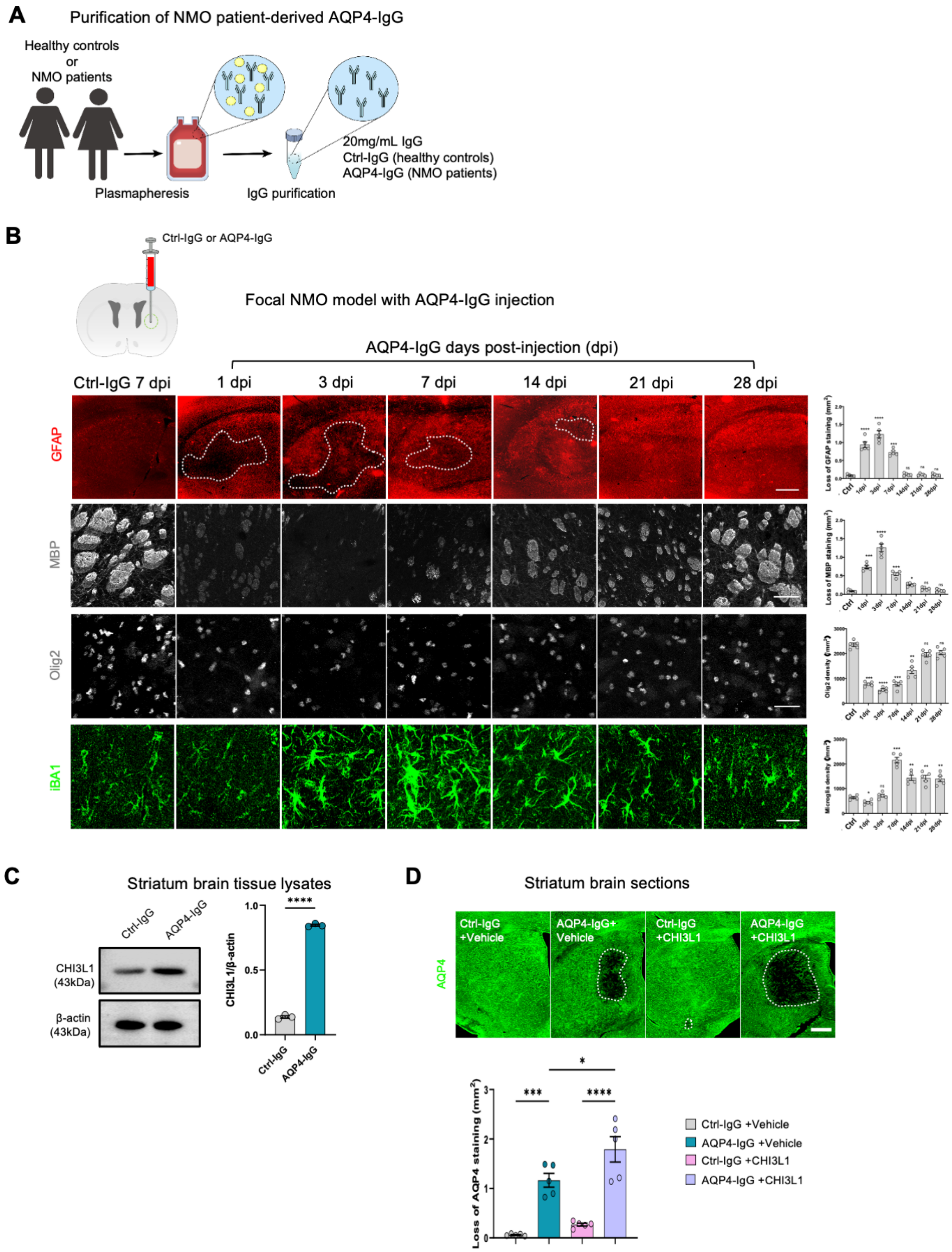

(A) Schematic illustration of the purification process for AQP4 autoantibodies (AQP4-IgG) and control IgG (Ctrl-IgG) from the sera of NMO patients and the control subjects of age- and sex-matched healthy donors, respectively.

(B) Time course (1–28 days post-injection, dpi) of focal striatal pathology after unilateral AQP4-IgG infusion (contralateral Ctrl-IgG as control) in 6-week-old WT mice: early astrogliosis (assayed by GFAP+ area), oligodendroglial loss (Olig2+ area), and demyelination (loss of MBP+ area) detectable at 1 dpi, peaking at 3 dpi; microgliosis (IBA1+ area) peaking at 7 dpi; partial recovery by 14 dpi and near-baseline by 28 dpi. $n = 5$ mice per group (3 sections per mouse). The 7-dpi time point was selected for downstream analyses.

(C) Immunoblot analysis demonstrating the induction of CHI3L1 expression in mouse striatal tissues following stereotaxic injection with AQP4-IgG or Ctrl-IgG in the focal NMO mouse model (7 dpi; related to **Fig. 1A-B**). Quantification is presented as the ratio of CHI3L1 densitometric intensity to the loading control β-actin. $n = 3$ mice per group.

(D) Confocal immunofluorescence images showing the internalization and degradation of AQP4 in the striatum induced by stereotaxic administration of AQP4-IgG and CHI3L1 (related to **Fig. 1A-B**). Areas with loss of AQP4 fluorescence signal are outlined with white dashed lines and quantified relative to the AQP4-IgG alone group (set to 1.0). Scale bars, 500 μm. $n = 5$ mice per group (3 sections per mouse).

All data are presented as mean ± SEM. For panel (**C**), statistical significance was evaluated using Student's *t*-test for two-group comparisons. For all other panels, statistical significance was assessed using one-way ANOVA with Tukey's post hoc multiple comparisons or Welch's ANOVA with Dunnett's T3 multiple comparisons test for data with unequal standard deviations. Non-significant comparisons are not indicated. * $p < 0.05$; ** $p < 0.01$; *** $p < 0.001$; **** $p < 0.0001$.

Figure S2. CHI3L1 amplifies spinal cord inflammation in NMO and directly impairs OPC proliferation and myelination (related to Fig. 1)

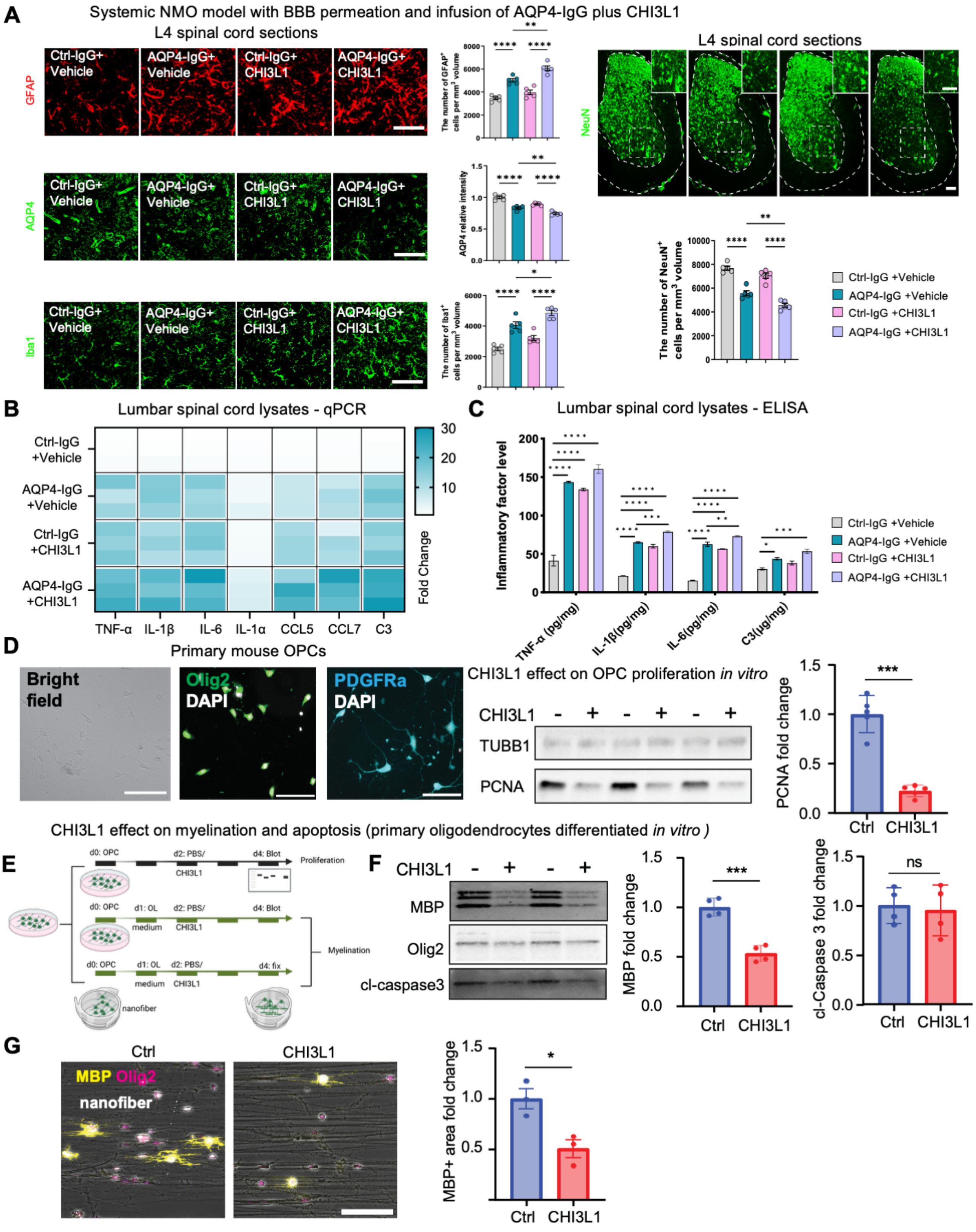

(A) Confocal immunofluorescence images depicting the effects of CHI3L1 on AQP4 loss, astrocyte activation ($GFAP^+$ cells), microglia activation ($Iba1^+$ cells), and ventral horn neuron loss ($NeuN^+$ cells) in L4 spinal cord sections from the systemic NMO mouse model (related to **Fig. 1G-I**). Quantifications include normalized AQP4 immunofluorescence signals (Ctrl-IgG + vehicle set to 1.0) and cell densities for each marker. Scale bars, 100 μm. $n = 5$ mice per group (3 sections per mouse).

(B) Quantitative PCR analysis of pro-inflammatory cytokine gene expression in the lumbar spinal cord of systemic NMO mice (related to **Fig. 1G-I**). mRNA levels of TNF-α, IL-1β, IL-6, IL-1α, CCL5, CCL7, and C3 were measured in lumbar spinal cord lysates from treated mice. Data are presented as a heat map with normalized values relative to the control condition (Ctrl-IgG + vehicle). $n = 3$ mice per group.

(C) ELISA measurements of pro-inflammatory cytokine protein levels in the lumbar spinal cord of systemic NMO mice (related to **Fig. 1G-I**). Secreted proteins of TNF-α, IL-1β, IL-6, and C3 were quantified in lumbar spinal cord lysates from treated mice. $n = 3$ mice per group.

(D) CHI3L1 reduces OPC self-renewal. Primary mouse OPCs were cultured and treated with CHI3L1 (500 ng/mL, 48 h). Left: representative images showing ramified, healthy OPC morphology expressing Olig2 and PDGFRα. Right: immunoblot for the proliferation marker PCNA with densitometric quantification. $n = 5$ independent experiments.

(E) CHI3L1 suppresses progression to myelinating oligodendrocytes without inducing cell death. Premyelinating oligodendrocytes (day 2 after induction) were exposed to CHI3L1 (500 ng/mL, 48 h) and analyzed by immunoblot for MBP (myelination marker) and cleaved caspase-3 (apoptosis control). $n = 4$ independent experiments.

(F) CHI3L1 impairs initiation of myelination on synthetic fibers. Differentiating oligodendrocytes were grown on aligned nanofibers and treated with CHI3L1 (500 ng/mL, 48 h); myelin ensheathment was quantified as MBP^+ area along fibers. $n = 3$ independent experiments.

All data are presented as mean ± SEM. For panel (D, F, G), statistical significance was evaluated using Student's *t*-test for two-group comparisons. For all other panels, statistical significance was assessed using one-way ANOVA with Tukey's post hoc multiple comparisons or Welch's ANOVA

with Dunnett's T3 multiple comparisons test for data with unequal standard deviations. Non-significant comparisons are not indicated. * $p<0.05$; ** $p<0.01$; *** $p<0.001$; **** $p<0.0001$.

Figure S3. Neutralizing CHI3L1 mitigates demyelination and CNS inflammation in *ex vivo* and *in vivo* NMO models (related to Fig. 2)

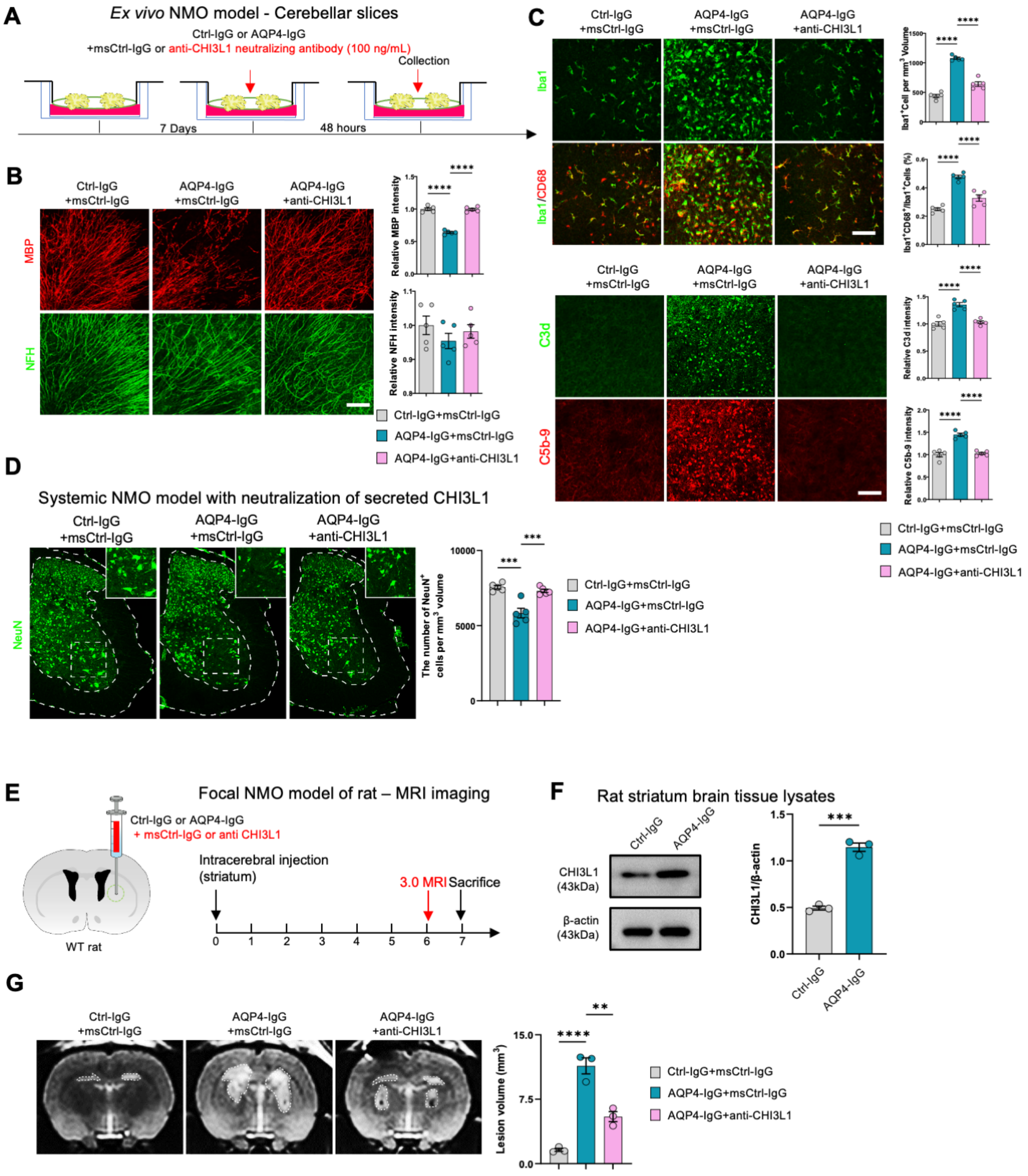

(A) Schematic diagram and experimental setup for the ex vivo NMO model using organotypic cerebellar slice cultures. Cerebellar slices from postnatal day 7 mice were cultured for 7 days. The slices were then treated for 48 hours with AQP4-IgG or Ctrl-IgG, along with either neutralizing anti-CHI3L1 antibody or control mouse IgG (msCtrl-IgG) at a concentration of 100 ng/ml. After treatment, the slices were immunostained for markers relevant to NMO pathology, focusing on myelination and glial activation.

(B) Confocal immunofluorescence images showing the effect of CHI3L1 neutralization on AQP4-IgG-induced demyelination in the *ex vivo* NMO model. Demyelination was quantified by measuring the fluorescence intensity of myelin basic protein (MBP) relative to the control condition (Ctrl-IgG + vehicle, set to 1.0). Neurofilament heavy chain (NFH) staining was used to assess the integrity of neuronal processes during the acute 24-hour treatment. Scale bars represent 100 µm. $n = 5$ slices per group (3 sections per slice).

(C) Confocal immunofluorescence images illustrating the effect of CHI3L1 blockade on microglial activation and complement activation in the *ex vivo* NMO model. Microglial activation was quantified by counting the cell density of total microglia ($Iba1^+$) and activated microglia ($Iba1^+/CD68^+$). Complement activation was assessed by measuring the levels of cleaved C3d, and membrane attack complex formation was evaluated by quantifying C5b-9 levels. All measurements were normalized to the control condition (Ctrl-IgG + vehicle, set to 1.0). Scale bars represent 100 µm. $n = 5$ slices per group (3 sections per slice).

(D) Confocal immunofluorescence images demonstrating the effects of CHI3L1 neutralization on AQP4-IgG-induced ventral horn neuron loss in L4 spinal cord sections of systemic NMO mice treated with anti-CHI3L1 or msCtrl-IgG (related to **Fig. 2A–G**). Neuronal loss was quantified by counting the density of $NeuN^+$ cells within the ventral horn region, with the Ctrl-IgG + vehicle group set to 1.0. Scale bars represent 100 µm. $n = 5$ mice per group (3 sections per mouse).

(E) Schematic representation and experimental timeline of the focal NMO rat model enabling MRI imaging of white matter changes. Young adult wild-type rats received stereotaxic injections into the striatum of either Ctrl-IgG or AQP4-IgG, along with co-administration of anti-CHI3L1 antibody or control msCtrl-IgG (6 µl of 1 mg/ml per animal). The rats underwent MRI 3.0T scans before being sacrificed for brain tissue collection.

(F) Immunoblot analysis showing CHI3L1 expression in rat striatal tissues after stereotaxic injection in the focal NMO rat model. Rats were injected with AQP4-IgG or Ctrl-IgG. Quantification is presented as the ratio of CHI3L1 densitometric intensity to the loading control β-actin. $n = 3$ rats per group.

(G) MRI examination evaluating white matter lesions in the injected striatum regions of the focal NMO rat model. Lesion volumes (in mm^3) were quantified in coronal striatum sections detected by 3.0 T MRI among the three indicated groups of rats. Lesion areas are outlined with white dashed lines. $n = 3$ rats per group.

Data are presented as mean ± SEM. For panel (F), statistical significance was evaluated using Student's *t*-test for two-group comparisons. For all other bar graphs, statistical significance was assessed using one-way ANOVA with Tukey's post hoc multiple comparisons or Welch's ANOVA with Dunnett's T3 multiple comparisons test for data with unequal standard deviations (SDs). Non-significant comparisons are not indicated. * $p<0.05$; ** $p<0.01$; *** $p<0.001$; **** $p<0.0001$.

Figure S4. Astrocyte-specific CHI3L1 deletion ameliorates NMO pathology in systemic NMO mice (related to Fig. 2)

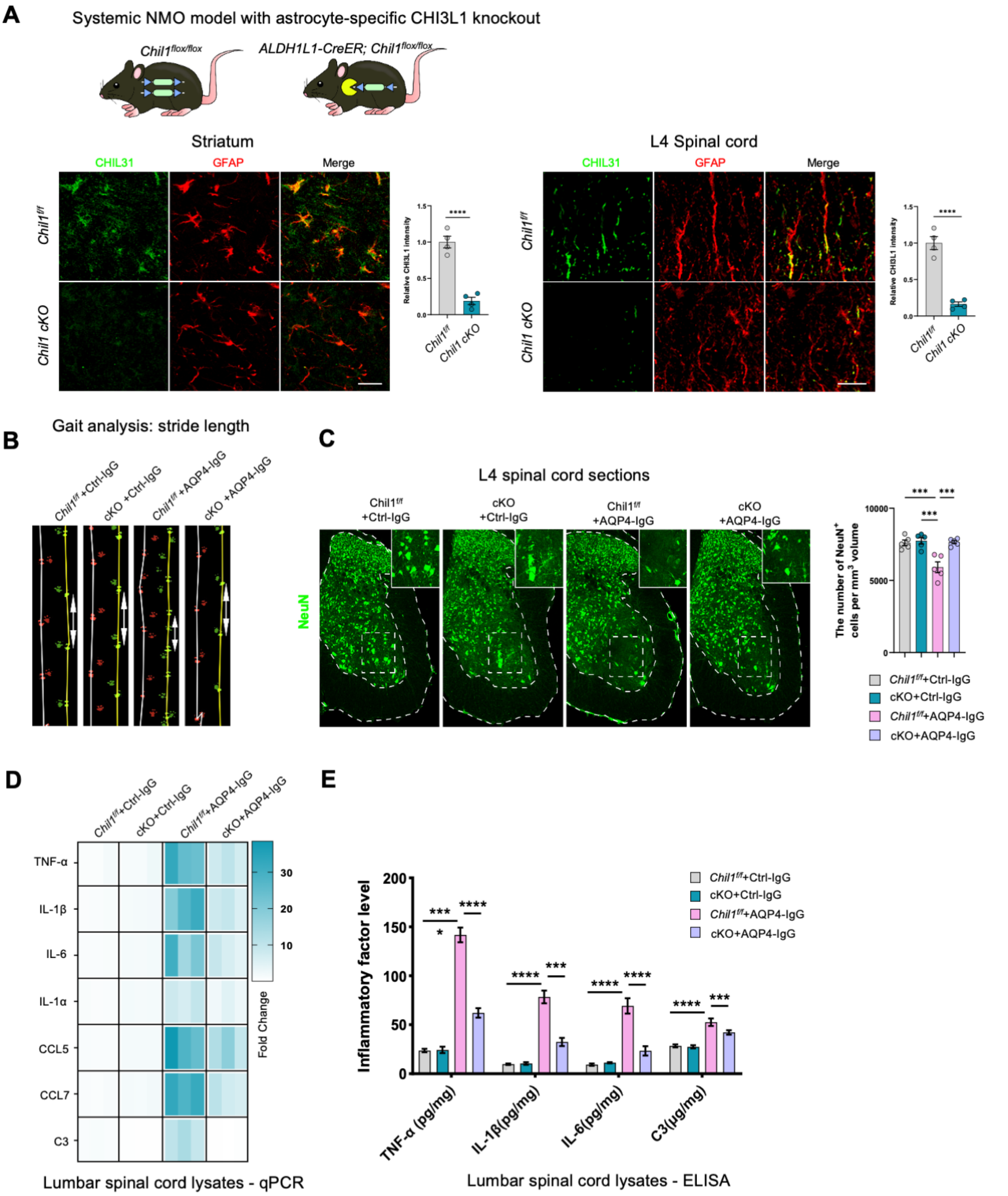

(A) Efficiency of CHI3L1 depletion in astrocytes confirmed in striatum and L4 spinal cord by IHC (~80% reduction in CHI3L1 signal in GFAP+ cells) in *Chil1* cKO versus *Chil1*$^{flox/flox}$ controls. *n* = 4 mice per group.

(B) Behavioral gait analysis assessing the effect of astrocyte-specific CHI3L1 deletion on stride length in systemic NMO mice (related to **Fig. 2I**). Stride length was measured to evaluate motor function, comparing systemic NMO mice with astrocyte-specific CHI3L1 deletion to control mice. *n* = 8 mice per group.

(B) Confocal immunofluorescence images showing the effect of astrocyte-specific CHI3L1 deletion on AQP4-IgG-induced ventral horn neuron loss in L4 spinal cord sections (related to **Fig. 2H–J**). Neuronal loss was quantified by counting the density of NeuN^{+} neurons within the ventral horn region. Scale bars represent 100 µm. *n* = 5 mice per group (3 sections per mouse).

(C) Quantitative PCR analysis of pro-inflammatory cytokine gene expression in the lumbar spinal cord of systemic NMO mice with astrocyte-specific CHI3L1 deletion (related to **Fig. 2H–J**). mRNA levels of TNF-α, IL-1β, IL-6, IL-1α, CCL5, CCL7, and C3 were measured in lumbar spinal cord lysates from treated mice. Data are presented as a heat map with values normalized to the control condition (Ctrl-IgG + vehicle). *n* = 5 mice per group.

(D) Quantification of secreted protein levels of inflammatory cytokines in lumbar spinal cord tissue lysates from systemic NMO mice with astrocyte-specific CHI3L1 deletion (related to **Fig. 2H–J**).

(E) Levels of TNF-α, IL-1β, IL-6, and C3 were measured using ELISA in tissue lysates from the four indicated groups of mice. *n* = 3 biological replicates per group.

Data are presented as mean ± SEM. For panel (A), statistical significance was evaluated using Student's *t*-test for two-group comparisons. For all other bar graphs, statistical significance was evaluated using one-way ANOVA with Tukey's post hoc multiple comparisons or Welch's ANOVA with Dunnett's T3 multiple comparisons test for data with unequal standard deviations. Non-significant comparisons are not indicated. * $p<0.05$; ** $p<0.01$; *** $p<0.001$; **** $p<0.0001$.

Figure S5. AQP4-IgG treatment upregulates NF-κB target genes in astrocytes (related to Fig. 3)

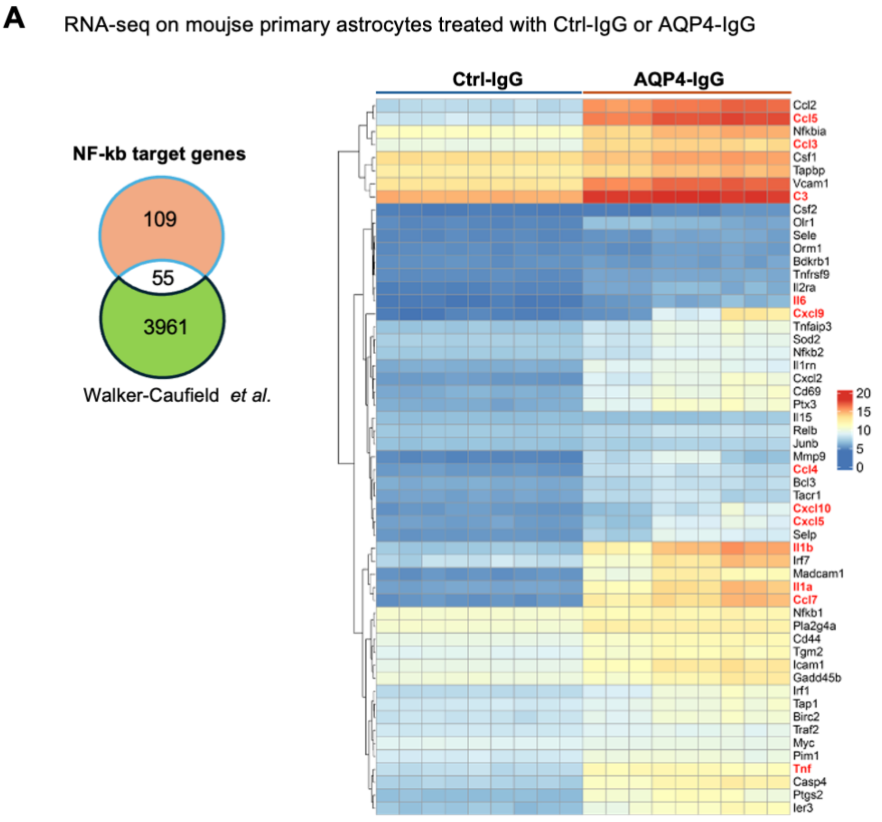

(A) We re-analyzed bulk RNA-seq data from primary mouse astrocytes exposed to AQP4-IgG or Ctrl-IgG (Walker-Caulfield et al., PMID: 26423139). Among 4,016 differentially expressed genes (DEGs), 55 overlapped with a curated set of 164 known NF-κB targets, indicating significant enrichment. A hierarchical heatmap displays $\log_2$ fold-changes for these 55 genes, normalized to the mean of untreated astrocytes ($\log_2FC = 0$; not shown), revealing uniform upregulation after AQP4-IgG treatment. Direct NF-κB transcriptional targets are highlighted in red.

Figure S6. Astrocyte-specific deletion of CHI3L1 receptor RAGE attenuates NMO Pathology by inhibiting CHI3L1-Induced NF-κB activation (related to Fig. 4)

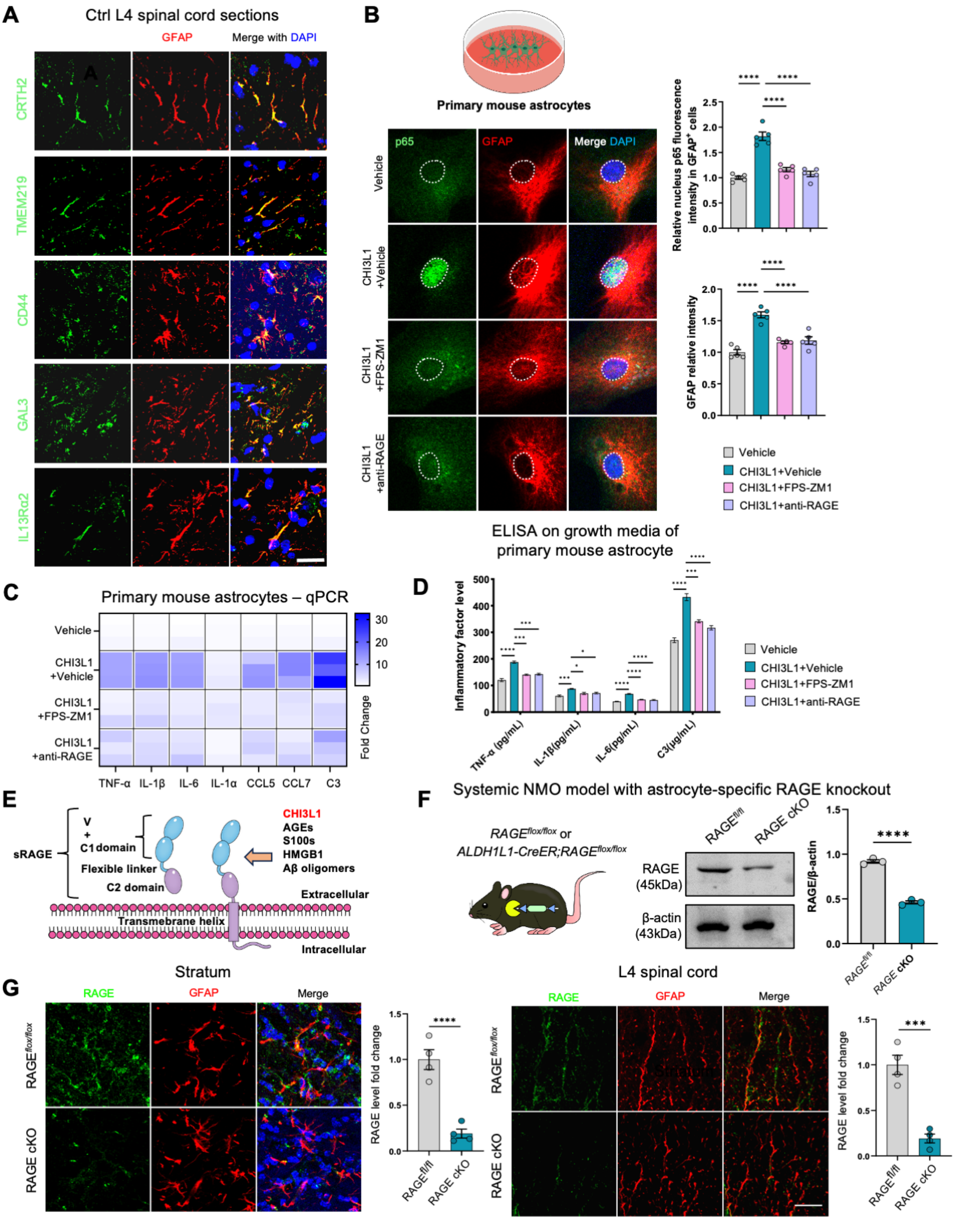

(A) Assessment of expression of individual CHI3L1 receptors in astrocytes in spinal cord, by immunofluorescence analysis of the expression of CRTH2, TMEM219, IL13Rα2, CD44, and Gal-3, and their colocalization with astrocytes marked by GFAP and DAPI within the L4 spinal cord section from control mice. Scale bar, 20 μm.

(B) Assessment of the effect of RAGE blockade on CHI3L1-induced NF-κB activation and astrocyte activation in astrocytes treated with AQP4-IgG and CHI3L1. Primary mouse astrocytes were treated with CHI3L1 (100 ng/ml) or vehicle, with the monoclonal anti-RAGE antibody to block ligand activation of RAGE (anti-RAGE, 100 ng/ml) or the selective RAGE inhibitor FPS-ZM1 (100 ng/ml) for 24 hours. Immunofluorescence analysis was performed to quantify nuclear localization of p65, expression levels of GFAP (astrocyte activation marker), plotted as relative to the control group (vehicle only =1.0). Scale bar, 20 μm. n=5 slides (3 sections/slide) per group.

(C) Quantitative PCR analysis of pro-inflammatory cytokine gene expression in astrocytes treated with vehicle or CHI3L1 (100 ng/ml) or vehicle with or without anti-RAGE (100 ng/ml) or FPS-ZM1 (100 ng/ml) for 6 hours. mRNA levels of TNF-α, IL-1β, IL-6, IL-1α, CCL5, CCL7, and C3 were measured. Data are presented as a heat map with values normalized to the vehicle control (=1.0). n = 3 experiments.

(D) Quantification of inflammatory cytokine protein levels secreted by astrocytes stimulated with CHI3L1 but with the RAGE function inhibited. Primary mouse astrocytes were treated with CHI3L1 at 100 ng/ml or vehicle, together with anti-RAGE (100 ng/ml) or FPS-ZM1 (100 ng/ml) for 24 hours. Levels of TNF-α, IL-1β, IL-6, and C3 in the culture media were measured using ELISA. n = 3 biological replicates per group.

(E) Schematic illustration of RAGE and its soluble form sRAGE. This panel depicts the protein structures of the receptor for advanced glycation end products (RAGE) and its cleaved soluble form, sRAGE. sRAGE functions as a decoy receptor by sequestering ligands, including advanced glycation end products (AGEs), S100 proteins, high mobility group box 1 protein (HMGB1), and amyloid-beta (Aβ) oligomers, thereby preventing them from binding to and activating membrane-bound RAGE. This inhibition blocks downstream pro-inflammatory signaling pathways.

(**F)** Immunoblot analysis showing RAGE depletion in lumbar spinal cord tissues from the transgenic mice with astrocyte-specific RAGE deletion by the administration of tamoxifen in the RAGE cKO mice (related to Fig. 4D-H). n=3 mice per group.

(G) Efficiency of astrocytic RAGE deletion confirmed by IHC in striatum and L4 spinal cord: ~80% reduction of RAGE signal within $GFAP^{+}$ cells in RAGE cKO mice versus $RAGE^{flox/flox}$ controls. n = 4 mice per group.

Bar graphs presented as the mean ± SEM; and statistical significance for all bar graphs but (F) was evaluated with one-way ANOVA and Tukey's post hoc multiple comparisons or Welch's ANOVA test with Dunnett's T3 multiple comparisons test for data with unequal standard deviation (SDs). For (F, G), Student's t-test for two-group comparisons. Nonsignificant comparisons were not identified. * p<0.05; ** p<0.01; *** p<0.001; **** p<0.0001.

Figure S7. RAGE blockade reduces demyelination and inflammation in NMO: clinical correlations with identified serum biomarkers (related to Fig. 4)

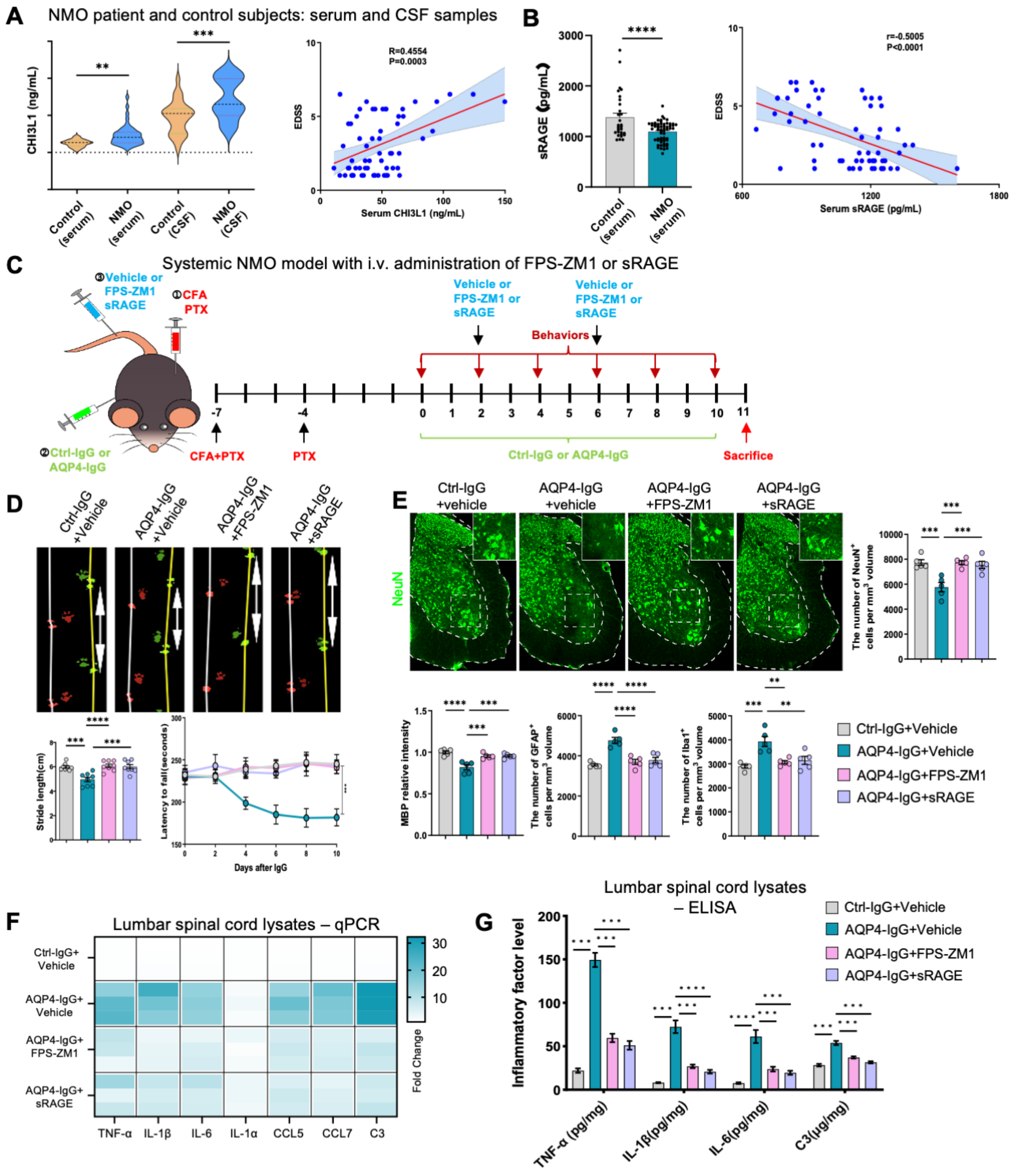

(A) CHI3L1 concentrations in the serum and cerebrospinal fluid (CSF) of NMO patients versus control subjects. CHI3L1 levels were determined by ELISA. A positive correlation was observed between CHI3L1 serum levels and Expanded Disability Status Scale (EDSS) scores in NMO patients, indicating that higher CHI3L1 protein levels are associated with increased neurological deficits as assessed by the EDSS. Sample sizes were: HC-serum, n = 50; NMO-serum, n = 60; HC-CSF, n = 30; NMO-CSF, n = 30.

(B) sRAGE concentrations in the serum of NMO patients versus control subjects. sRAGE levels were measured by ELISA. A negative correlation was found between sRAGE serum levels and EDSS scores in NMO patients, suggesting that lower sRAGE levels are associated with greater neurological impairment as evaluated by the EDSS. Sample sizes were: HC-serum, n = 50; NMO-serum, n = 60.

(C) Schematic and experimental design to evaluate the effect of RAGE blockade on NMO pathology. Using the systemic NMO mouse model, mice were treated with sRAGE (10 μg/mouse) or the RAGE antagonist FPS-ZM1 (10 μg/mouse) to assess their effects on disease progression and inflammatory responses.

(D) Behavioral assessments evaluating the effect of RAGE inhibition on motor dysfunction in systemic NMO mice. Gait analysis was used to quantify stride length, and the Rotarod test measured latency to fall. These assessments were performed on four groups: Ctrl-IgG + vehicle, AQP4-IgG + vehicle, AQP4-IgG + sRAGE, and AQP4-IgG + FPS-ZM1. n = 8 mice per group.

(E) Confocal immunofluorescence images showing the effects of RAGE inhibition on AQP4-IgG-induced demyelination, glial activation, and neuronal loss. Quantifications include the relative fluorescence intensity of myelin basic protein (MBP) to assess demyelination, and cell densities of $GFAP^+$ astrocytes (astrogliosis), $Iba1^+$ microglia (microgliosis), and $NeuN^+$ neurons (neuronal loss) in L4 spinal cord sections. Scale bars represent 100 μm. n = 5 mice per group (3 sections per mouse).

(F) Quantitative PCR analysis of pro-inflammatory cytokine expression in the lumbar spinal cord of systemic NMO mice following RAGE inhibition. mRNA levels of NF-κB target genes, including TNF-α, IL-1β, IL-6, IL-1α, CCL5, CCL7, and C3, were measured in lumbar spinal cord lysates. Data are presented as a heat map with values normalized to the control condition (Ctrl-IgG + vehicle = 1.0). n = 3 mice per group.

(G) Quantification of pro-inflammatory cytokine protein levels in lumbar spinal cord tissue lysates. Levels of TNF-α, IL-1β, IL-6, and C3 were measured using ELISA in tissue lysates from systemic NMO mice treated with RAGE inhibitors and control groups. n = 3 mice per group.

Data are presented as mean ± SEM. For panels (E), (F), and (H), statistical significance was evaluated using one-way ANOVA with Tukey's post hoc multiple comparisons test, or Welch's ANOVA with Dunnett's T3 multiple comparisons test for datasets with unequal standard deviations (SDs). For panels (B) and (C), Student's t-test was used for two-group comparisons. Correlations between EDSS scores and CHI3L1 or sRAGE serum levels in panels (B) and (C) were analyzed using Pearson correlation coefficients. For the latency to fall in the Rotarod test in panel (E), a two-way ANOVA was used. Significance levels: * $p<0.05$; ** $p<0.01$; *** $p<0.001$; **** $p<0.0001$. Non-significant comparisons are not indicated.

Figure S8. Activation of the JAK-STAT3 signaling pathway in astrocytes activated by AQP4 autoantibodies (related to Fig. 5)

A

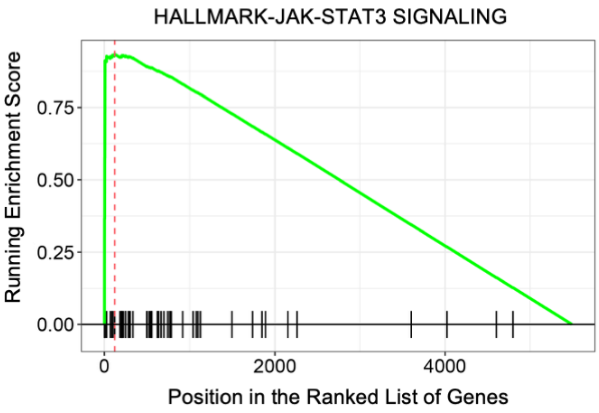

B

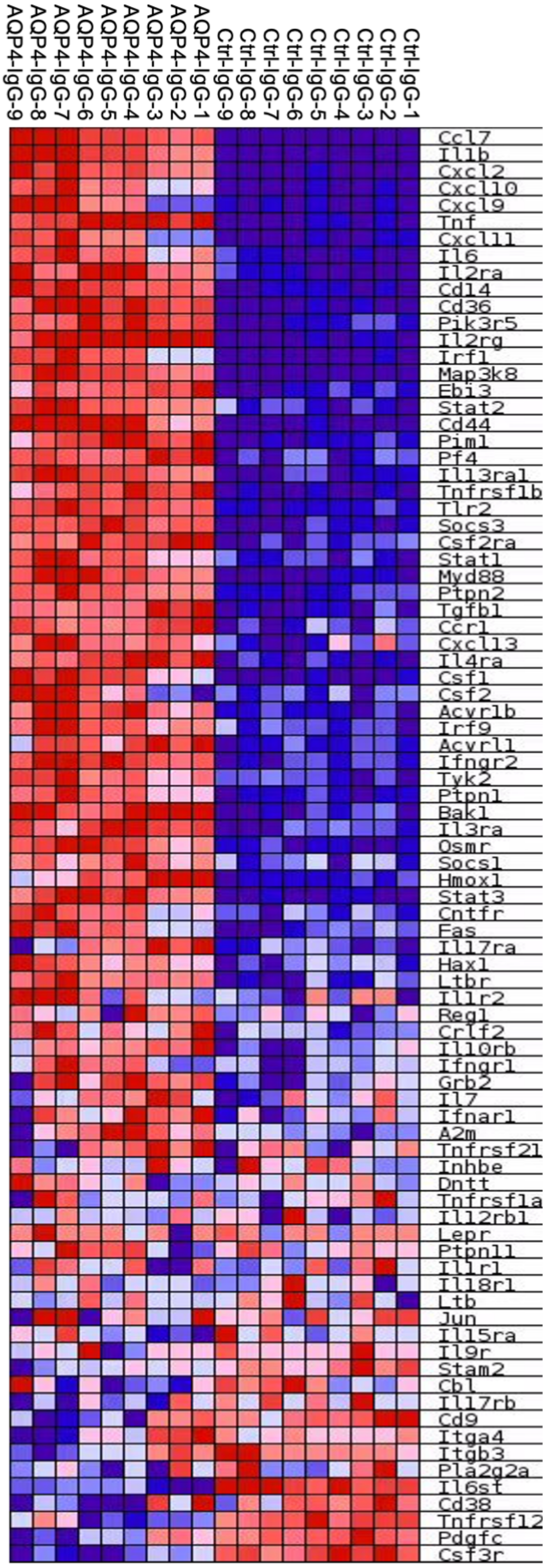

(A) Gene Set Enrichment Analysis (GSEA) revealing activation of the JAK-STAT3 signaling pathway in astrocytes treated with AQP4-IgG. We conducted GSEA on the differentially expressed genes (DEGs) identified from our RNA-seq analysis of primary mouse astrocytes treated with AQP4-IgG versus control IgG (Ctrl-IgG). The analysis demonstrates significant enrichment of gene sets related to the JAK-STAT3 signaling pathway, indicating its activation upon AQP4-IgG treatment (related to **Fig. 5A**).

(B) Comparison of JAK-STAT3 pathway-related DEGs between our study and Walker-Caulfield et al. Transcriptomic analysis identified 85 DEGs associated with the JAK-STAT3 signaling pathway that overlap between our dataset and the published dataset by Walker-Caulfield et al. (PMID: 26423139). This overlap underscores the consistent activation of the JAK-STAT3 pathway in astrocytes upon exposure to AQP4 autoantibodies (related to **Fig. 5A**).

Figure S9. Astrocyte-Selective STAT3 deletion mitigates AQP4-IgG–induced pathology in both the focal and systemic NMO models (related to Fig. 5)

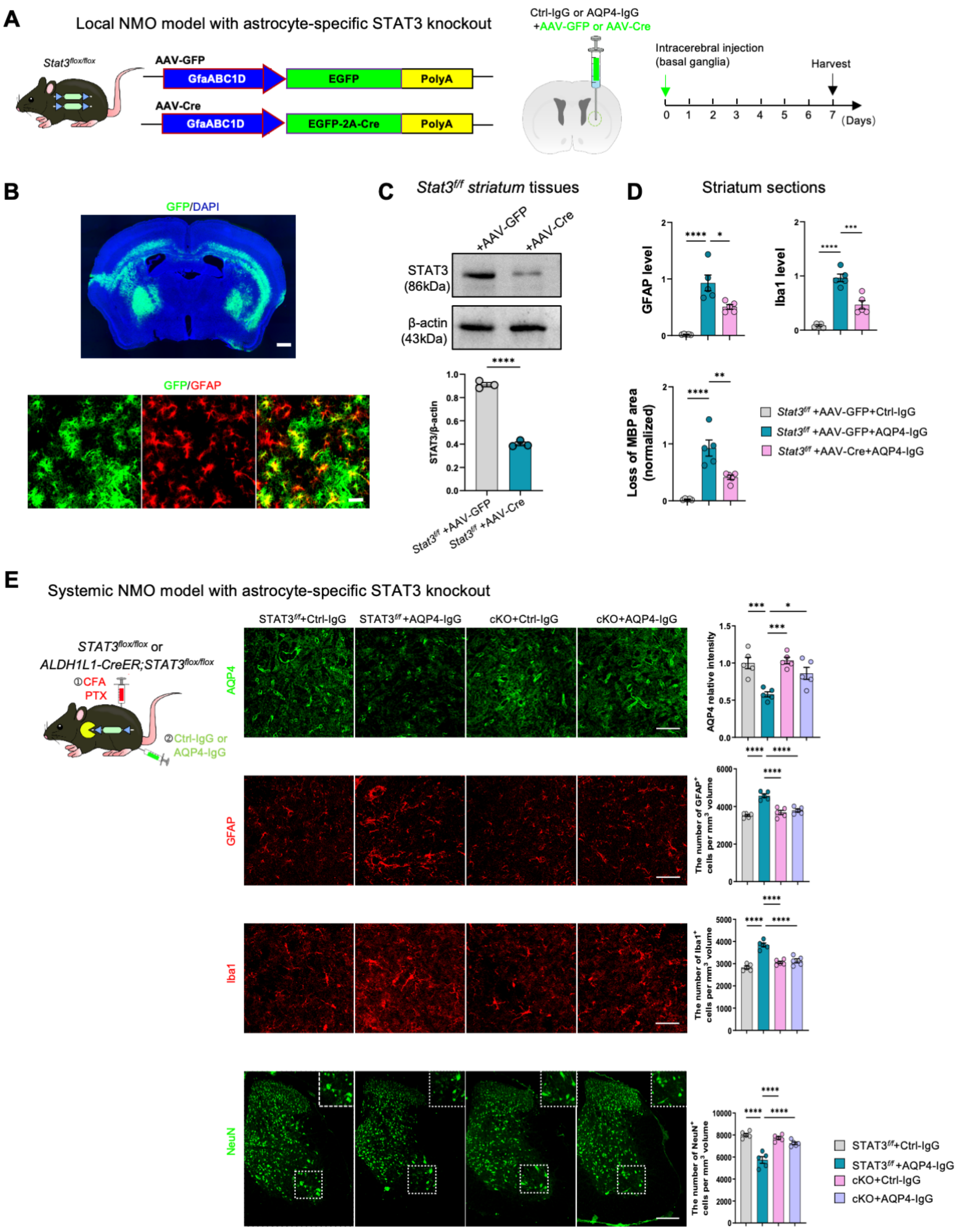

(A) Schematic representation and experimental timeline of the focal NMO mouse model with conditional astrocyte-specific STAT3 deletion in the striatum. To achieve inducible astrocyte-specific knockout of STAT3, STAT3-floxed transgenic mice (*STAT3$^{f/f}$*) were combined with an AAV-based approach. Mice received stereotaxic injections into the striatum of either Ctrl-IgG or AQP4-IgG, along with AAVs expressing GFP-tagged Cre recombinase (AAV-Cre) or GFP alone (AAV-GFP) under the control of the astrocyte-specific GfaABC1D promoter.

(B) Assessment of the specificity and efficiency of astrocyte-specific STAT3 deletion. Immunofluorescence analysis was conducted in the AAV-injected striatum region to evaluate GFP expression (co-expressed with Cre) in astrocytes marked by GFAP. This confirmed the specificity and efficiency of AAV-mediated Cre recombinase transduction in astrocytes. Scale bars: 500 μm (upper panel) and 20 μm (lower panel).

(C) Evaluation of STAT3 depletion efficiency via AAV-mediated Cre recombination. Immunoblotting analysis was performed on striatal tissue lysates from *STAT3$^{f/f}$* mice that received stereotaxic injections of AAV-GFP on one side and AAV-Cre on the other side of the striatum. Densitometric analysis was plotted as the ratio of STAT3 to the loading control β-actin. n = 3 mice per group.

(D) Confocal immunofluorescence images illustrating the effects of astrocyte-specific STAT3 deletion on AQP4-IgG-induced pathology in the striatum. The images show demyelination (assessed by loss of MBP-stained area), astrocyte activation (GFAP signal), and microglial activation (Iba1 signal) within the injected striatum region. Quantifications include the relative immunofluorescence intensity, normalized to the condition of *STAT3f/f* + AAV-Cre + AQP4-IgG. n = 5 mice per group (3 sections per mouse).

(E) IHC assessment of astrocyte-specific STAT3 deletion on NMO pathology in the systemic model. Representative immunohistochemistry showing that conditional deletion of STAT3 in astrocytes mitigates AQP4-IgG–induced lesions. Top three rows (striatum): restoration of AQP4 signal, reduced astrogliosis (GFAP) and decreased microgliosis (Iba1). Bottom row (L4 spinal cord): preservation of ventral-horn neurons (NeuN). Quantifications were derived from the mean of 3 sections per animal; n = 5 mice per group.

Data are presented as mean ± SEM. For panel (C), statistical significance was evaluated using Student's t-test for two-group comparisons. For all other bar graphs, statistical significance was assessed using one-way ANOVA with Tukey's post hoc multiple comparisons test, or Welch's ANOVA with Dunnett's T3 multiple comparisons test for datasets with unequal standard deviations (SDs). Non-significant comparisons are not indicated. Significance levels: * $p<0.05$; ** $p<0.01$; *** $p<0.001$; **** $p<0.0001$.
