# Supplementary material for: Astrocyte-intrinsic signaling of chitinase-like protein CHI3L1 drives inflammation and amplifies demyelination in neuromyelitis optica
Source: J Clin Invest. 2026 Jan 2;136(1):e195506. doi: 10.1172/JCI195506 (PMC12721897; doi:10.1172/JCI195506)

Figure 1C

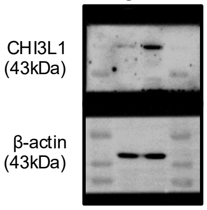

Figure S1B

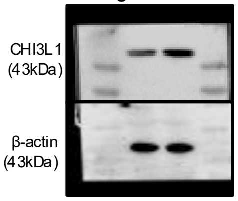

Figure 3B

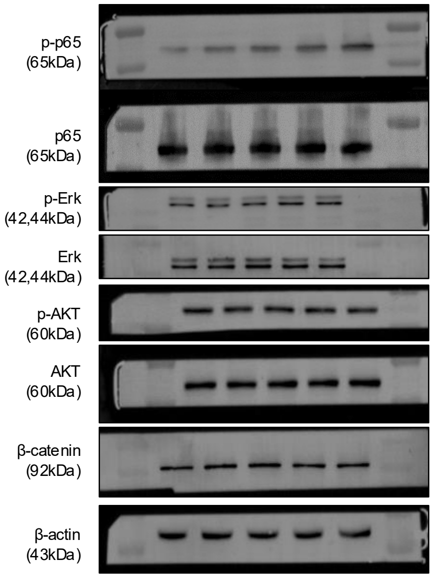

Figure 3F

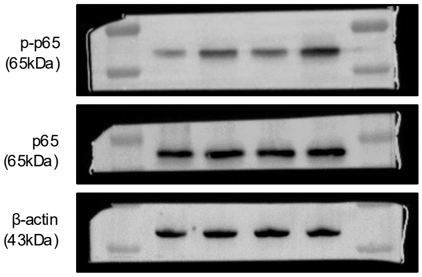

Figure 4C（Left）

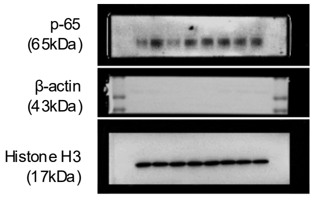

Figure 4C (Right)

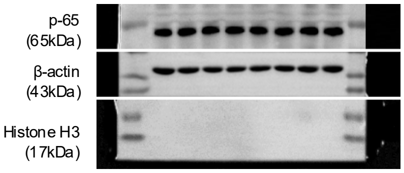

Figure S6E

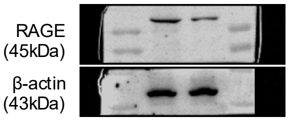

Figure 5B

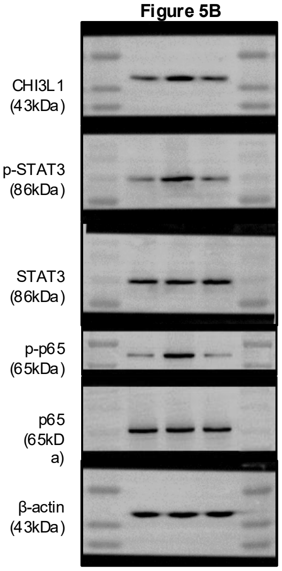

Figure 5C

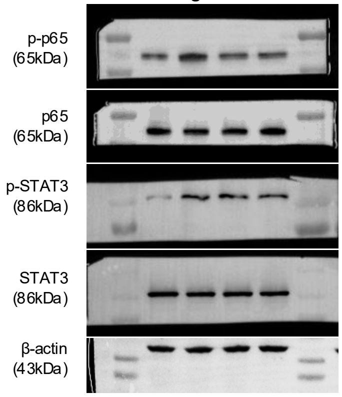

Figure 6E

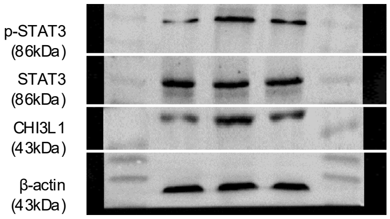

Figure S9D

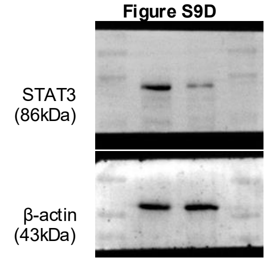

Figure S2F

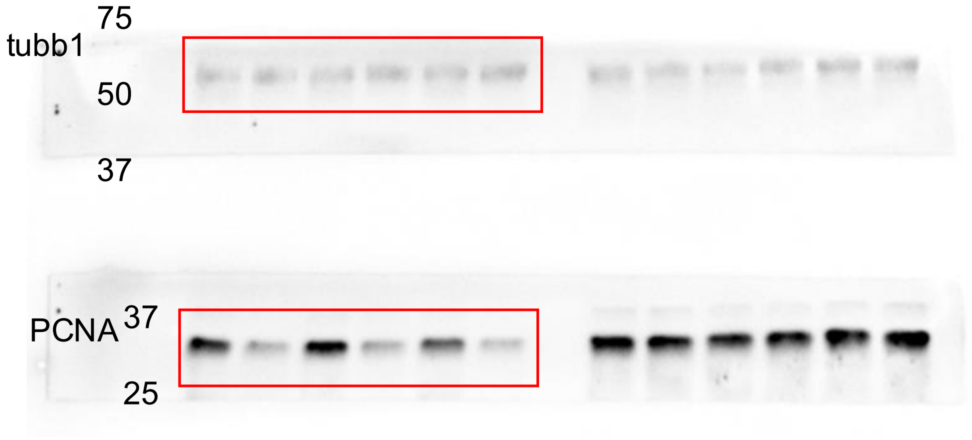

Figure S2G

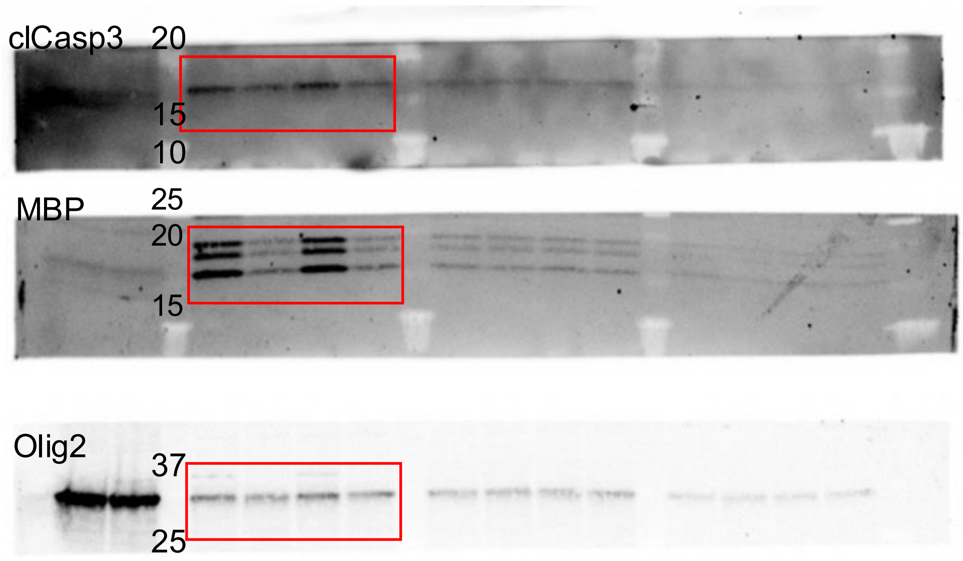

Supplement: Unedited blot and gel images [file jci-136-195506-s305.pdf]
